# Myelin development in the peripheral nervous system of *Trachemys scripta*

**DOI:** 10.3389/fcell.2026.1810247

**Published:** 2026-06-18

**Authors:** Ricardo Lara-Ramírez, Nikolas Yousefi, Martin van der Plas, Mayra Sánchez-Hernández, Monserrat Suárez-García, Carolina Martínez-López, Alejandra Valdés-Aviña, Rodolfo Daniel Ávila-Avilés, Torben Ruhwedel, Wiebke Mobius, Maria Elena de Bellard

**Affiliations:** 1 Centro de Investigación en Ciencias Biológicas Aplicadas (CICBA), Facultad de Ciencias, Universidad Autónoma del Estado de México, Toluca, Mexico; 2 Biology Department, MC8303, California State University Northridge, Northridge, CA, United States; 3 Centro Conjunto de Investigación en Química Sustentable (CCIQS), UAEM-UNAM, Toluca, Mexico; 4 Electron Microscopy-City Campus, Department of Neurogenetics, Max Planck Institute for Multidisciplinary Sciences-City Campus, Göttingen, Germany

**Keywords:** MPZ, myelin, peripheral nerve, reptile, Schwann cell

## Abstract

**Introduction:**

Myelin is one of the most important features of the vertebrate nervous system, formed by glial cells. In the peripheral nervous system, Schwann cells produce myelin. While much is known about the molecules and processes involved in mammalian myelination, little is known about it in other vertebrate groups.

**Methods:**

In this study, we examined myelin development in the peripheral nerves of the turtle Trachemys scripta using qPCR, RNA sequencing, immunofluorescence, and TEM.

**Results:**

Our findings indicate that in T. scripta, myelination begins during the late stages of embryonic development and continues beyond hatching. Expression profiles reveal both conservation and divergence of core myelination components between mammals and amphibians; however, direct gene-level comparisons across species require further investigation given our small sample size

**Discussion:**

The upregulation of orthologous myelin genes in the turtle PNS supports the idea that these components are conserved, although their timing, regulation, or network structure may differ across tetrapods. We based our inference of conservation on orthology and the coordinated expression of key myelin genes (e.g., ErbB2, Mag, Mpz, Mbp, Pmp22) in T. scripta. TEM analysis of turtle sciatic nerves shows myelin rings in a few axons starting at stage 21, increasing significantly by stage 24, and becoming prominent in most axons of the adult nerve. Furthermore, antibodies against MPZ, DRP2, and Kv1.1 indicate that T. scripta adult peripheral nerves at various axial levels contain myelin segments with structures consistent with appositions and Schmidt-Lanterman incisures. Overall, this is the first study of PNS myelin development in a reptile, demonstrating that myelination is a highly conserved process in vertebrates.

## Introduction

The vertebrate peripheral nervous system includes sensory neurons and glial cells. The primary peripheral glia is the Schwann cell, which is responsible for axon survival and myelination (Brügger et al., 2023; Ceci et al., 2014; Jessen and Mirsky, 2019). Myelination is a highly advantageous adaptation that evolved at different times in various animal groups (de Bellard, 2016; Weil et al., 2018; Zalc, 2016). The first chordates to show the presence of compact myelin are gnathostomes, since no compact myelin has been detected in hagfish or lampreys (Bullock et al., 1984; Zalc, 2006).

PNS myelination involves the extensive wrapping of Schwann cell plasma membranes around an axon, excluding the cytoplasm and leaving only the plasma membrane (Castelfranco and Hartline, 2015; Garbay et al., 2000). In the vertebrate PNS, this process begins during late embryonic development when Schwann cells start expressing key myelin genes, but it mainly occurs after birth in mammals (Baron et al., 1994a; Baron et al., 1994b). Initially, Schwann cells wrap around multiple peripheral axons and then sort them until there is a one-to-one ratio between Schwann cells and axonal segments (Jessen and Mirsky, 2019; Mirsky et al., 2008). The vertebrate PNS is unique in possessing Remak bundles (Monk et al., 2015), which are groups of axons from C nociceptive fibers that remain unmyelinated and bundled by the wrapping of Schwann cells (Murinson et al., 2005). Besides this developmental peculiarity, PNS myelin consists of a specific, highly conserved set of glycoproteins that are essential for maintaining the structural stability of myelin segments, and this set is conserved across mammals (Boyle et al., 2001; Einheber et al., 1997; Eshed et al., 2005).

Interestingly, myelination has independently evolved in various metazoan lineages, including annelids, crustaceans, and all vertebrates except agnathans (Castelfranco and Hartline, 2015; Gould et al., 2008; Hartline, 2008; Hartline and Colman, 2007; Zalc, 2016). While most of our knowledge about myelin structure and development comes from studies on mammals, it remains unclear whether the key molecules involved in mammalian myelination serve similar functions in other vertebrates. Additionally, the timing of PNS myelination across vertebrate groups has not yet been established. Although the basic mechanisms and molecules involved in peripheral myelin development in reptiles are believed to be highly conserved, further research is necessary to fill species-specific gaps and to fully understand the diversity and adaptation of myelination within this vertebrate group.

Studying myelin formation and structure in reptiles can offer valuable insights into the evolution of this important vertebrate trait, since this group of animals sits between amphibians and mammals and has existed on Earth for 230 million years (Cleary et al., 2020). Because turtles are a basal sauropsid lineage with a unique evolutionary history, they serve as an ideal model for exploring the ancestral features of amniote myelination (Lee et al., 2020), thereby expanding our understanding of nervous system evolution in this group. Considering turtles’ evolutionary position, we hypothesize that their myelin will be similar to that of other land vertebrates such as mammals.

Here, we analyzed the expression of homologs of mammalian genes involved in PNS myelination in developing peripheral nerves of the turtle *Trachemys scripta*. We used qPCR, RNA sequencing, and transmission electron microscopy (TEM) to evaluate changes in myelination during embryonic development. Our qPCR results for specific genes involved at different stages of myelination indicate that myelination peaks late in the turtle’s embryonic development and concludes after hatching. Additionally, we also examined *T. scripta* peripheral nerves by TEM at various embryonic stages. We observed that myelin formation is already quite advanced in hatchlings but remains low in pre-hatching embryos, suggesting that hatching marks a stage comparable to mammalian birth in the onset of myelination. Finally, we studied the cellular structure of myelin segments in different adult *T. scripta* peripheral nerves. We found that they have unique myelin specializations, including structures consistent with Schmidt-Lanterman incisures and myelin appositions. Overall, our data expands our understanding of myelin development and structure beyond mammals and indicates shared molecular and cellular features of myelin in amniotes.

## Results

### RNA-seq in *T*. *scripta* embryos reveals differentially expressed genes related to myelination

Murine PNS myelination begins embryonically by E18, with MPZ mRNA expression increasing and peaking by P10 (Baron et al., 1994a; Baron et al., 1994b). To identify a somewhat equivalent embryonic stage in turtles, we selected an embryo that still does not look like a developed turtle (st.19) and one that already shows color and appears developed (st.21) (Supplementary Figure S1) (Noravian et al., 2025). We chose these two developmental stages, given their external morphology, to collect PNS nerves that could correspond to postnatal days 0–10 in mammals.

We started our study of PNS myelination in *T. scripta* by conducting a preliminary RNA sequencing (RNA-seq) analysis of peripheral nerves from embryos at stages 19 (N = 1) and 21 (N = 1). Although the sample size could not be statistically significant (N = 1), we found some interesting differences in gene expression between these stages. Principal Component Analysis (PCA) showed a distinct separation between st.19 and st.21, with PC1 accounting for 87.4% of the variance (Supplementary Figure S2A), indicating major transcriptional changes between these two stages. The PCA plots further supported this distinction (Supplementary Figure S2B).

**FIGURE 1:**
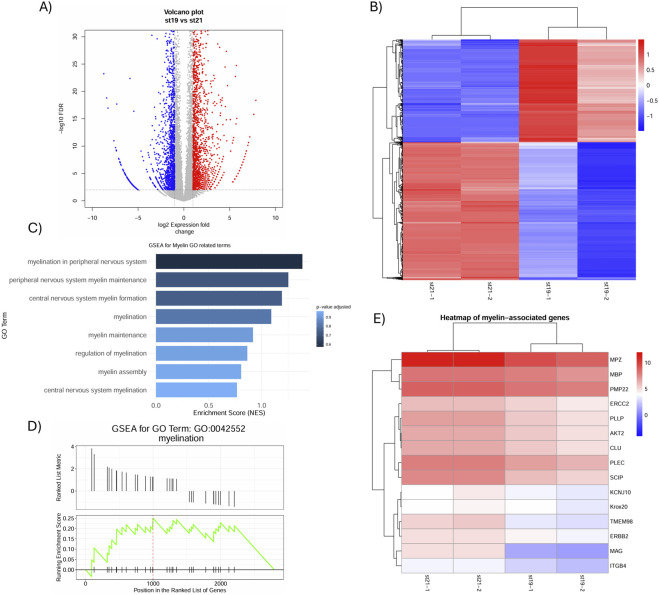
Differential expression analysis (DEG) between st.19 and st.21. **(A)** Volcano plot and **(B)** Heatmap plot of differentially expressed genes on RNA-seq on peripheral nerve tissues collected from embryos at developmental stages 19 (st.19, N = 1) and 21 (st.21, N = 1). **(C)** Gene Set Enrichment Analysis (GSEA) for myelin related gene ontologies (GO), and **(D)** GSEA analysis for myelination GO (GO:0042552). **(E)** Heatmap plot of myelin associated genes on RNA-seq on peripheral nerve tissues collected from embryos at developmental st.19 and st.21.

Differential expression analysis (DEG) between st.19 and st.21 identified 2,181 upregulated and 1,630 downregulated genes in st.21 compared to st.19 (see Supplemental Excel file with all the DE genes). A volcano plot (Figure 1A) highlights these differences. To further explore gene expression across samples, heatmaps were generated, with genes (rows) normalized to ensure consistent representation across samples, and clustering was applied to both genes and samples. The heatmap of DEGs (Figure 1B), based on FPKM values, shows distinct clustering patterns that reflect possible real transcriptional changes between stages and account for biological replicates within each condition.

Since we were examining myelination development, we analyzed the expression patterns of key genes. A targeted Gene Set Enrichment Analysis (GSEA) focusing on GO terms related to myelination (Figure 1C) revealed significant enrichment, with each GO term represented by its Normalized Enrichment Score (NES) and adjusted p-value. Among these, the GO term myelination showed a strong correlation with differentially expressed genes, as illustrated in the enrichment plot (Figure 1D), indicating coordinated regulation of myelination-associated pathways between st.19 and st.21. To visualize these changes better, we generated a heatmap of myelination-related genes (Figure 1E). The heatmap highlights a general increase in MPZ, MBP, PMP22, ERBB2, and MAG at st.21 compared to st.19. The small size of the nerve samples made dissection difficult, which could have led to contamination from surrounding tissues and potentially affected the gene expression results.

Gene Ontology (GO) analysis was performed separately for upregulated and downregulated genes to better understand the functional implications of these changes in gene expression. The analysis revealed that genes upregulated in st.21 were significantly enriched in biological processes related to biosynthesis and metabolism, including cellular biosynthetic processes, protein metabolism, and organonitrogen compound metabolism. Additionally, enrichment in developmental pathways, such as anatomical structure development, multicellular organism development, and cell differentiation, suggests increased cellular specialization and tissue organization at this stage (Supplementary Figure S3A). Conversely, genes downregulated in st.21 were mostly associated with cellular component organization and biogenesis, nervous system development, and neurogenesis. The downregulation of microtubule-based movement, cilium organization, and growth regulation indicates decreased cytoskeletal dynamics and cellular extensions, reflecting a shift toward cellular stabilization and functional maturation (Supplementary Figure S3B). Overall, these findings suggest that increased biosynthetic and developmental activities characterize st.21. At the same time, processes related to structural reorganization and neural differentiation are relatively downregulated, possibly indicating a transition in cellular function and energy utilization.

### Expression of myelin-related genes in *Trachemys scripta* peripheral nerves suggests early and late phases of myelination

After identifying key myelin genes from our st.19 and st.21 transcriptome data, we expanded the developmental range to stages 12–25 in *T. scripta* embryos to analyze myelin gene expression by qPCR. Our phylogenetic analyses showed that *T. scripta* possesses orthologs of *ErbB2*, *Itgb*, *Krox20*, *Scip/Oct-6*, *Mag*, *Mbp*, *Mpz*, and *Pmp* (Supplementary Figures S4–S11).

**FIGURE 2:**
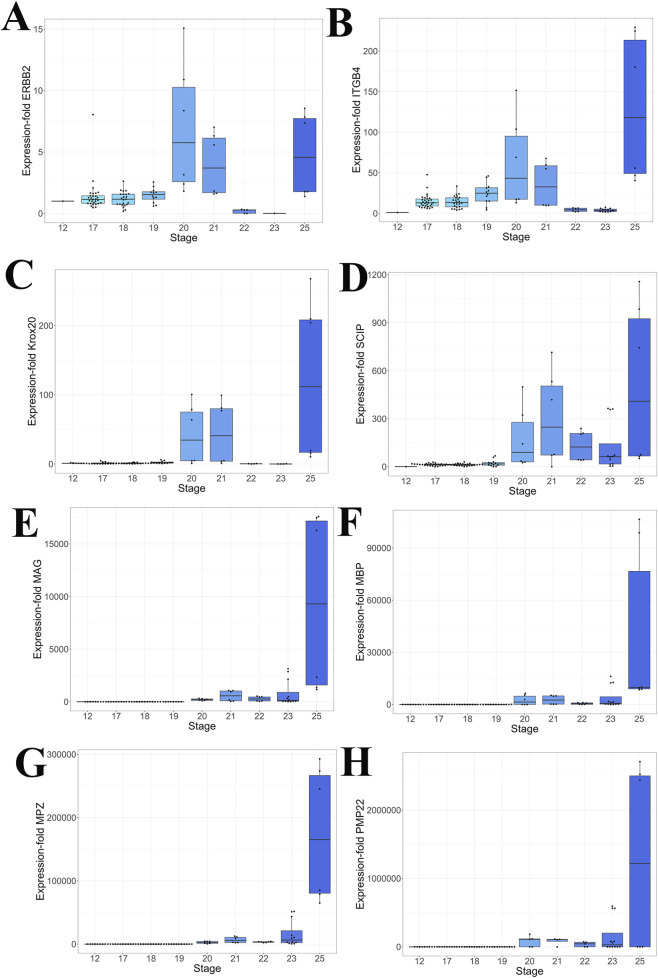
Relative expression levels of myelin genes in *T. scripta* peripheral nerves across development. Expression fold changes of myelination marker genes from qPCR experiments are shown for each developmental stage in samples with two or more biological replicates. The graphs show the expression fold of each of the following markers: **(A)** *ErbB2*, **(B)** *Itgb4*, **(C)** *Krox20*, **(D)** *Scip*, **(E)** *Mag*, **(F)** *Mbp*, **(G)** *Mpz*, **(H)** *Pmp22*. The level of expression of each marker at each stage is relative to the base expression (stage 12). Only samples with two or more biological replicates are shown in these graphs. Expression-fold change was calculated using the Livak method/delta-delta Ct method (2^(-ΔΔCt)^). Gene organization, calculations for ΔCt, ΔΔCt, and the Livak method, and statistical analyses were performed in Microsoft Excel. Graphing was done using RStudio. Statistics: Data were log-transformed, and normality was checked using the Shapiro-Wilk test. An ANOVA was used for parametric data, with a Tukey test applied if p < 0.05. A Kruskal–Wallis test was used for non-parametric data, with pairwise Wilcoxon tests if p<*p < 0.05, **p < 0.01, ***p < 0.001. See Supplementary Table S2 for sample sizes for each stage, ranging from N = 1 to N = 5.

Here, we examined the expression of these genes in the combined sciatic and brachial nerves of developing turtle embryos (Figure 2). These developmental stages cover the formation of spinal nerves (st.12) through the hatching phase (st.25) (Cordero and Janzen, 2014) with a sample size ranging from N = 1 to N = 5 for each stage (Supplementary Table S2). However, some of the st.22-23 samples for *Krox20* and *erbB2* were excluded from the analysis due to low qPCR yields. Expression levels of myelin genes were normalized to *GADPH* and shown as delta CT values, following the standard qPCR graphing method outlined by Livak (Livak and Schmittgen, 2001). Examination of the *ErbB2* receptor and *Itgb4*, well-known receptors involved in peripheral nervous system myelination (Van der Zee et al., 2008), revealed increased expression during stages 20–21 and 25, compared to lower levels during stages 12–19 and 22–23 (Figures 2A,B). Similarly, *Krox20*, a transcription factor involved in the early phases of myelination (Decker et al., 2006; Ghislain et al., 2003), showed a comparable expression pattern to *ErbB2* and *Itgb4* (Figure 2C). Expression of *Scip*, another key transcription factor crucial in the transition from pre-myelinating to myelinating Schwann cells (Lemke et al., 1991; Monuki et al., 1990), was lower during stages 12–19 and peaked at st.25, reaching approximately 49 times higher than the basal level at st.12 (Figure 2D). All these differences were statistically significant. Overall, these results suggest that the initial stages of Schwann cell differentiation into myelinating glia in *T. scripta* peripheral nerves begin at st.20, and the bimodal gene expression profile indicates two waves of gene activity.

We also analyzed the expression of key myelin genes by qPCR. The expression patterns of *Mag, Mpz, Mbp, and Pmp22* in turtle embryos were similar and, unlike those of genes involved in myelin initiation in mammals, they showed a single expression peak at st.25. These genes displayed minimal expression during stages 12–19 and low expression at stages 20–23 (Figures 2E–H). However, their expression increased notably by st.25 (∼5,000- and 200,000-fold higher than basal expression). These results suggest that the first steps in myelination begin quite early, at stages 20–23 in peripheral nerves of *T. scripta*, while the structural proteins necessary for compact myelin begin to peak by stage 25.

### Ultrastructure of turtle peripheral nerves during development

**FIGURE 3:**
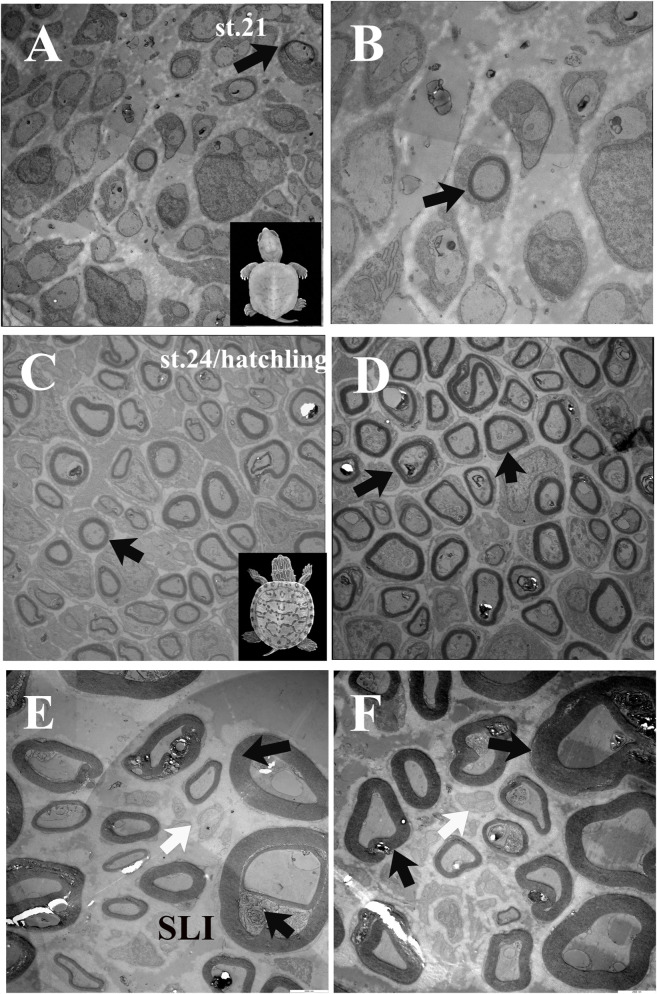
Transmission electron microscopy of *T. scripta* peripheral nerves during development. Sections of the sciatic nerves at different developmental stages. **(A,B)** St.21 nerves present scant myelin rings. **(C,D)** St.24/hatchling nerves contain considerably more myelinated axons, although myelin rings are still thin. **(E,F)** Adult nerves contain several well-myelinated axons, with thick myelin rings. White arrows in **(E,F)** show Remak bundles. Black arrows in **(E,F)** show thick myelin segments. Photographs of embryonic specimens are shown in the insets.

Because our combined RNA sequencing and qPCR data showed that *T. scripta*’s myelin genes peak at stage 25, and because immunofluorescence staining for MPZ and MBP is absent during nerve development across hatching (see below), we dissected nerves (brachial and sciatic plexuses) from embryos between st.21 (N = 1 per stage) and one adult for transmission electron microscopy (TEM) to examine compact myelin (Figure 3). TEM analysis of turtle nerve embryos confirmed that by st.21, most nerves lack compact myelin, except for one nerve showing signs of the myelination process (arrows in Figures 3A,B). However, by st.24/hatchling phase, most nerves contain significantly more myelinated axons, although the myelin rings remain thin, indicating ongoing myelination (arrows in Figures 3C,D). The myelin thickness appears similar regardless of axon size, suggesting incomplete myelination in turtle nerves at hatching. TEM sections of adult *T. scripta* nerves revealed thicker compact myelin rings around large-caliber axons and thinner myelin around narrower axons (black arrows in Figures 3E,F), as well as Remak bundles consisting of unmyelinated small nerves, likely C-fibers. These bundles are enveloped by non-myelinating Schwann cells, resembling what is seen in mammalian nerves (white arrow in Figure 3F). Because of the small sample size, the above ultrastructural assessments are qualitative. We can only state that myelination in turtle peripheral nerves begins before hatching but remains incomplete until adulthood.

### *T*. *scripta* adult nerves possess myelin specializations like those of mammals

**FIGURE 4:**
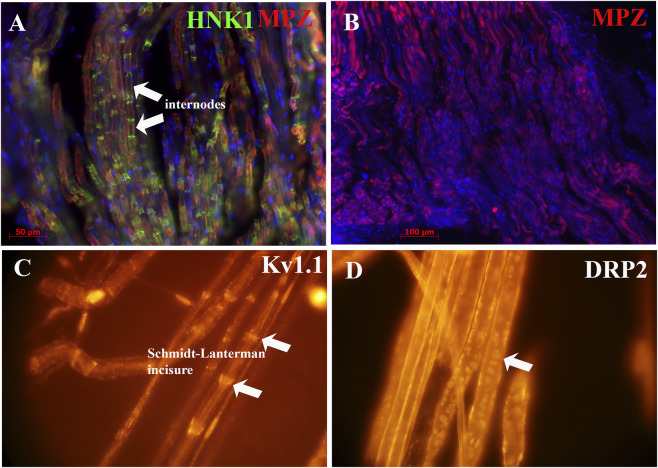
Immunostaining of peripheral nerves in *T. scripta* adults. **(A,B)** Sections of the sciatic nerve stained with antibodies against MPZ highlight myelin internodes. Antibodies against HNK-1 carbohydrates reveal the presence of structures similar to Schmidt-Lanterman incisures (arrows in A). **(C)** Staining for the potassium channel Kv1.1 in the cervical nerve reveals its presence in structures consistent with Schmidt-Lanterman incisures (arrows). **(D)** Appositions in the cervical nerve are visualized with antibodies against DRP2 as small patches of staining distributed irregularly along myelin internodes.

To determine whether the qPCR data showing increased *Mpz* and *Mbp* could be confirmed by immunostaining, we next examined various st.21–24 peripheral nerves. However, we did not observe any positive staining for MPZ or MBP in st.21 or st. 24 nerves (Supplementary Figure S12). We then co-stained adult nerves with antibodies against MPZ and HNK1 (Figures 4A,B), a marker known for its expression in myelin (Martini et al., 1994; Pedraza et al., 1995). We observed robust staining with MPZ along nerves, while HNK1 labeled distinct regions within myelin segments that resembled Schmidt-Lanterman incisures (white arrows in Figure 4A) (Martini and Schachner, 1986).

To further identify myelin specialization in different *T*. *scripta* adult peripheral nerves, we examined the expression of MPZ to highlight internodes (Supplementary Figures S13,A,D,G,J,M) and of the potassium channel Kv1.1, known to be present in juxtaparanodes, Schmidt-Lanterman incisures, and the inner mesaxon (Arroyo et al., 1999). Our analysis showed Kv1.1 staining in regions with a trapezoidal shape and regular spacing, similar to the pattern observed in Schmidt-Lanterman incisures (Figure 4C; Supplementary Figures S13C,F,I,L,O). The Kv1.1 expression pattern closely resembled that of HNK1 (Figure 4A). In mammals, peripheral myelin features Cajal bands and appositions at the outer myelin layer. DRP2 is a protein involved in adhesion at these appositions (Sherman et al., 2001; Sherman et al., 2012). Using antibodies against human DRP2 in *T. scripta* adult peripheral nerves, our immunofluorescence experiments revealed patches of staining on the outer surface of myelin segments across all nerves examined, resembling patterns seen in mammalian myelin appositions (Figure 4D; Supplementary Figures S13B,E,H,K,N). Notably, the shape and distribution of these appositions varied among turtle nerves, with thoracic nerves showing the sparest pattern. These results suggest the presence of myelin structures consistent with specializations corresponding to appositions and Schmidt-Lanterman incisures described in mammals.

## Discussion

We provide the first detailed description of PNS myelination in the turtle *T. scripta*. Our findings indicate that PNS myelination begins *in ovo* and continues after hatching. This includes the first RNA sequencing data from *T. scripta* peripheral nerves. From a morphological point of view, structures consistent with Schmidt-Lanterman incisures and appositions were observed in adult *T. scripta* myelin segments, structures previously known mainly from mammals. Together, our results show that turtle myelin shares both conserved and unique features compared to mammalian myelin.

### Myelination in *T. scripta* likely begins around stages 19–21 and is transcriptionally prominent by stage 25; structural maturation continues post-hatching

MPZ mRNA and protein expression in rat peripheral nerves begins prenatally (around embryonic day 18) and rises sharply during the early postnatal period, coinciding with the onset and progression of myelination (Baron et al., 1994a; Baron et al., 1994b). However, there is no presence of compact myelinated nerves in the embryonic mammalian PNS (Capodivento et al., 2024; Stassart et al., 2013). Our findings show that in *T. scripta* PNS nerves, MPZ and other myelin genes are highly expressed, and we also observed compact myelin in some embryonic nerves (Figures 2, 3).

Our limited transcriptomic data on myelin development in *T*. *scripta* showed a shift of key myelin genes between st.19 and 21. Differential expression of myelination-related GO terms (Figures 1C,D), and peak expression of compact myelin genes at st.25 (Figures 2E–H) converge with TEM evidence (Figure 3) to strengthen the case for a conserved process of myelination in *T. scripta*.

Studies in other reptiles also show when myelination begins. In the lizard *Eumeces fasciatus*, myelination in the CNS starts before hatching, with strong PLP expression (Nadon and Duncan, 1995). Myelin rings were observed in TEM sections of the dorsal funiculus of the cervical spinal cord since E21 of *E*. *fasciatus* development up into adulthood. Although the authors did not inspect the PNS, the general trend of myelin formation is similar to that observed in *T. scripta* peripheral nerves, with myelination starting *in ovo* and detectable by gene expression and myelin sheath identification. Together, these data indicate that, in reptiles, myelin formation starts during *in ovo* embryonic stages, and progresses after hatching, in contrast to mammals, whose PNS myelination is mostly postnatal (Fraher, 1976; Zeiss, 2021). However, although adulthood was used as a clear benchmark for fully mature myelin ultrastructure in this species, we acknowledge a limitation: intermediate juvenile time points were not examined, preventing a precise determination of when myelination reaches adult-like completeness.

### Adult *Trachemys scripta* peripheral nerve myelin is very similar to that of mammals

In our immunofluorescence data, we observed that DRP2 and Kv1.1 proteins in *T*. *scripta* peripheral nerves matched canonical mammalian distributions (Sherman et al., 2001; Sherman et al., 2012). All five turtle peripheral nerves studied showed compact myelin (marked by MPZ) and myelin appositions (marked by DRP2). To our knowledge, myelin appositions have been described only in mammals, and our results constitute the first report of these myelin specializations outside mammals. The presence of DRP2 in turtles’ myelinated nerves suggests a similar adhesion system at myelin appositions.

Our findings on the distribution patterns of Kv1.1 and HNK1, which likely highlight the presence, distribution, and shape of what appear to be Schmidt-Lanterman incisures in turtle PNS myelin segments, suggest a conserved function akin to their mammalian counterparts. Similar incisures have also been reported in axolotl (Uckermann et al., 2019) and chicken (Tetzlaff, 1982), indicating that they are a conserved feature in tetrapod PNS myelin. Thus, adult turtle PNS myelin segments display DRP2, Kv1.1, and HNK1 patterns consistent with appositions and Schmidt-Lanterman incisures similar to those in mammals (Arroyo et al., 1999).

## Conclusion

Based on pooled brachial and sciatic nerve qPCR and sciatic nerve immunostaining, *T*. *scripta* myelination likely begins around stages 19–20. Our data indicate that turtles express orthologs of canonical mammalian PNS myelin genes (e.g., *ErbB2, Mag, Mpz, Mbp, and Pmp22*) during development, consistent with Schwann cell–mediated PNS myelination. These expression profiles highlight the conservation and divergence of core myelination components across mammals and reptiles. Comparative transcriptomics and protein localization in adults support conservation of myelination genes in turtles, although functional requirements were not tested here. Based on gene expression levels and TEM observations, the myelination process begins *in ovo* and continues after hatching. Adult turtle PNS myelin segments exhibit patterns consistent with appositions and Schmidt-Lanterman incisures, similar to mammals.

## Methods

### Animal collection and nerve tissues

Adult female specimens of *Trachemys scripta* were collected from the CSUN campus terrain. They were taken to the animal care facility, where they were injected with 1 mL of oxytocin (Kawazu et al., 2014) to induce oviposition, and then returned to the local pond. The collected eggs were placed in a container with 20 g of vermiculite and 20 mL of water, thoroughly mixed, and sealed with a lid to prevent evaporation. Each container only held eggs from a single batch and was labeled with the oviposition date. The containers were kept in an incubator at 28 °C. The lids were opened weekly to refresh the air in the container and inspect the eggs. Moldy eggs were removed and discarded. The developmental stage of the embryos was determined following Greenbaum’s protocol (Greenbaum, 2002). Embryos at different developmental stages were collected.

A total of 31 specimens of *T. scripta* were euthanized according to the CSUN IACUC-approved Animal Protocol. Thirty collected specimens were embryos, ranging from stage 12 (st.12) to st.26, and one specimen was sacrificed 4 days post-hatching. The developmental stage of each embryo was determined using literature (Cordero and Janzen, 2014). Parts of the brachial and sacral plexus (nerves) were collected since individual nerves were too small to provide enough RNA. Half of the collected tissue from each specimen was stored in 4% paraformaldehyde (PFA) at 4 °C and half in RNA-later at −20 °C.

### RNA extraction and cDNA synthesis

Total RNA was extracted from embryonic tissues stored in RNA-later using the Thermo Genejet RNA Purification Kit. RNA concentrations from *T. scripta* limb samples, nerve samples, and whole embryos ranged from 6.4 to 58.1 ng/μL, 0–16.9 ng/μL, and 15.6–17.8 ng/μL, respectively. The RNA concentration was measured using the Thermo Scientific NanoDrop 2000. A fraction of the total RNA was reverse transcribed into cDNA using the Thermo SuperScript VILO cDNA Synthesis Kit following the manufacturer’s instructions and stored at −20 °C.

### RNA sequencing and data analysis

High-quality samples, selected based on quality control (QC) assessments, were sequenced at Arraystar (Rockville, MD, United States). In addition, a batch of peripheral nerve tissue from *Trachemys scripta* was prepared for RNA sequencing at CSUN using the Illumina TruSeq Stranded mRNA LT Sample Prep Kit. The integrity of the RNA was confirmed by running the products on a 2% TBE agarose gel, and DNA concentrations were quantified using a Thermo Fisher Qubit 2.0 fluorometer to assess the success of the preparation. The generated cDNA libraries were also used for subsequent qPCR analysis. Genome and annotation files for *T. scripta* used in the RNA-sequencing analysis were downloaded from the NCBI RefSeq assembly GCF_013100865.1 for *T. scripta elegans* (Brian Simison et al., 2020). Index files were constructed using Hisat2.

RNA-seq data from *Trachemys scripta* peripheral nerves collected at embryonic st.19 and st.21 were processed and analyzed using the Rsubread 2.16.1, edgeR 3.42.4, and limma 3.56.2 packages in R. The sequencing data, consisting of single-end BAM files, were aligned to the *T. scripta elegans* genome downloaded from the NCBI RefSeq assembly GCF_013100865.1 (Brian Simison et al., 2020), and gene counts were obtained using featureCounts with the genome annotation in GTF format. Raw count data were imported into edgeR, and genes with low expression were filtered by excluding those with counts per million (CPM) less than 1 in fewer than two samples. Data normalization was performed using calcNormFactors to account for differences in sequencing depth, followed by dispersion estimation using estimateDisp. A design matrix was created to compare the st.19 and st.21 groups. Principal component analysis (PCA) and multidimensional scaling (MDS) were conducted to assess sample similarity and variance in gene expression. Differential gene expression analysis was performed using glmFit and glmLRT in edgeR, with a contrast comparing the st.19 and st.21 groups. Differentially expressed genes (DEGs) with a log fold change (logFC) greater than 1 and a false discovery rate (FDR) less than 0.01 were considered upregulated, while those with logFC less than −1 and FDR less than 0.01 were considered downregulated. These DEGs were exported for further analysis.

A Volcano plot was generated using logFC and FDR values. Genes with upregulated expression were highlighted in red, while downregulated genes were shown in blue, with the remaining genes in gray. Thresholds of |logFC| > 1 and FDR <0.05 were applied to identify significantly differentially expressed genes. Next, the RPKM/FPKM and TPM values were calculated for the RNA-seq data. RPKM/FPKM values were transformed to a log2 scale, and average values were computed for both stages. Log2 TPM values were then calculated to visualize gene expression patterns across samples. Heatmaps were generated using the pheatmap 1.0.12 library in R for gene expression visualization. The rows (genes) were normalized to ensure consistent representation across samples, and clustering of both genes and samples was applied. A color scale ranging from blue to red indicated low to high expression, respectively.

Gene Ontology (GO) enrichment analysis was performed to identify biological processes associated with differentially expressed genes (DEGs) identified in the RNA-seq data. The analysis was conducted separately for upregulated and downregulated genes using ShinyGO v0.741 (Ge et al., 2020), focusing on the Biological Process (BP) ontology. A significance threshold of p-value <0.05 was applied, and the results were used to determine enriched functional categories within each gene set.

Additionally, a specific analysis explored GO terms related to myelin-associated processes. Key GO terms of interest were selected from the enrichment results, and graphical representations were generated. This included bar plots showing the enrichment scores for each selected term, along with their significance and adjusted p-values. To further illustrate the impact of gene expression changes on myelination, enrichment plots were generated for the GO term myelination (GO:0042552), providing insights into the transcriptional shifts occurring between developmental stages.

### Primer design and qPCR

The genes selected for qPCR were *Itgb4, Krox-20, Mag, Mbp, Mpz, Pmp22,* and *Scip*, as they are expressed at different stages of the myelination process (Gerber et al., 2021; Jessen and Mirsky, 2019; Rouillard et al., 2016). *ErbB2* was used as a positive control, and the housekeeping gene *GAPDH* was used to normalize the Ct values. Using the BLAST primer tool, the sequencing results were used to design primers that generated amplicons of 100–150 bp (Supplementary Table S1). Nineteen turtle embryo samples were selected for qPCR based on RNA concentration and developmental stage (Supplementary Table S2). To set the base expression, two st. 12 embryos were used as a calibrator for the qPCR experiment. A total of 1,026 qPCR reactions were run, of which 70 did not give a Ct value. There were two or more biological replicates for stages 12, 17–19, and 23 embryos. There was only one embryo at stages 20–22 and 25. The cDNA pool was created with eight combined samples. A second primer set was used to perform nested PCR on the amplified products from the first primer set. Nested PCR products were run on a 1% TAE agarose gel and extracted using the Thermo GeneJET Gel Extraction Kit. The extracted products were sent to Laragen (Culver City, CA, United States) for sequencing. A selection of cDNA samples was used for qPCR. Each sample was run in triplicate on two separate plates, giving six technical replicates per sample. The ThermoFisher Power Up SYBR Green protocol was used on a Bio-Rad CFX96 touch real-time PCR detection system. A total of 40 cycles were run with an annealing temperature of 52 °C. The results of all reactions were combined in a single Excel file. We visualized expression dynamics across stages using fold-change plots based on the ΔΔCt method (ΔCt (calibrator). This was calculated following the Livak method (2001). The data were further processed and analyzed using R Studio version 1.0.153. Data were log-transformed, and normality was checked using the Shapiro-Wilk test. An ANOVA was used for parametric data, with a Tukey test applied if p < 0.05. A Kruskal–Wallis test was used for non-parametric data, with pairwise Wilcoxon tests if p < 0.05. Reactions with stage 23 samples using primers for *Krox20* and *erbB2* were excluded from the analysis because only a fraction of the qPCR reactions yielded results. For all markers except *erBb2*, the data were non-parametric.

### Transmission electron microscopy

Dissected samples from stage 21, 24, and adult brachial and sciatic plexuses were immersed in primary fixative (Karlsson-Schultz phosphate buffer: 109.5 mM NaH_2_PO_4_·H_2_O, 93.75 mM Na_2_HPO_4_.2H_2_O, 86.2 mM NaCl, 2.5% glutaraldehyde, 4% formaldehyde, pH 7.4) and processed as described previously (Weil et al., 2018). The samples were rinsed three times for 15 min with 0.1 M phosphate buffer at 4 °C. Samples were post-fixed for 4 h with 2% OsO_4_ at 4 °C and rinsed thrice with H_2_O for 15 min each at 4 °C. After several washes with H_2_O, the samples were dehydrated through ascending acetone concentrations for 15 min each (30%, 50%, 75%, 90%, 3 %× 100%). The samples were incubated at increasing resin concentrations (2:1, 1:1, 1:2) for 2 h each, then left in 100% EPON overnight. The next day, the samples were incubated with fresh 100% EPON for 4 h and polymerized for 24 h at 60 °C. Ultrathin sections of embedded nerve samples were cut using an ultramicrotome (RMC PowerTome PT-PC, Science Services, Munich, Germany) and a 35° diamond knife (Diatome, Biel, Switzerland). Sections were placed on 100 mesh hexagonal copper grids (Science Services) and imaged with a LEO912 electron microscope (Carl Zeiss Microscopy, Oberkochen, Germany) and an on-axis 2 k CCD camera (TRS, Moorenweis, Germany).

### Immunofluorescence

For embryonic nerves, immunofluorescence was performed using our lab’s previous protocols (Goldberg et al., 2020) and modified as follows: 5 embryo sections were placed in a well containing 1,000 μL of blocking solution [5% Goat serum/1% Triton-X 100] and incubated for 24 h at 4 °C. Once permeabilized, 500 μL of the blocking solution was removed, and 1 μL of the primary antibody, acetylated Tubulin (Sigma-Aldrich mouse IgG2b isotype) or HNK1 supernatant at a 1:10 dilution, was added to the well to yield a 1:500 dilution. The plate containing the samples was placed on an agitator set to the lowest speed for 24 h at 20 °C–22 °C. Samples were then incubated at 4 °C for 24 h. Sterile-filtered 1X PBS was used to wash all the samples. A total of 3 washes were performed using 1,000 μL of 1X PBS for each exchange, with 4 exchanges per wash. Sections remained in the solution for 30 min during each wash. The solution in the well was then reduced to 500 μL, and 1 μL of the conjugate secondary antibody, Alexa Fluor™ 488 goat anti-mouse (Invitrogen, Thermo Fisher Scientific), and 10 μL of the DAPI/PBS solution were added. The well was placed on the agitator at the lowest setting for 1 h at 20 °C–22 °C. Wells were then washed using the previously described method. The immunostained sections were placed on a glass slide with 1X PBS and a coverslip. Samples were observed under a Zeiss AxioImager A1 microscope, photographs were taken with an AxioCam camera, and images were processed using AxioVision 4.8 software. Photos were taken with the 4X, 10X, and 20X objectives. The acetylated Tubulin sections were imaged using 3 channels.

Adult nerve immunofluorescence of peripheral nerves was previously described (Lara-Ramírez et al., 2008). Facial, cervical, thoracic, brachial, and sciatic plexus nerves were teased with fine needles onto slides coated with poly-L-lysine and left to air-dry for 1 h. Samples were later fixed with 4% PFA for 20 min and washed three times in PBS for 5 min each. Samples were incubated in 10% non-fat skimmed milk powder in PBS (blocking solution) for 1 h at 4 C. Primary antibodies against MPZ (polyclonal IgG rabbit anti-MPZ, Novus), Kv1.1 (monoclonal IgG rabbit anti-Kv1.1, Novus), and DRP2 (polyclonal rabbit anti-DRP2, Novus) were then added at a 1:50, 1:100, and 1:50 concentration in PBS, respectively, and samples were incubated overnight at 4 °C. Negative controls for the primary Ab were carried out and expression was negative (data not shown). We also checked the epitope sequence across many vertebrates, and the antibody amino acid sequence was strongly conserved (Supplementary Figure S14). Samples were then washed three times in PBS for 5 min each and then incubated with the anti-IgG secondary antibody conjugated with Cy3 (polyclonal goat anti-rabbit IgG, Novus) at a 1:100 concentration for 1 h at 4 °C. Nerves were then washed three times in PBS for 5 min each and later mounted in non-fluorescent aqueous medium (UltraCruz). Samples were visualized under an AxioLab A1 microscope and photographed using a Canon camera at different magnifications.

### Phylogenetic analyses

Sequences of myelin-related genes were searched in genomes of representative species of the main vertebrate groups (Supplementary Table S4) in the NCBI and Ensembl public databases. Sequences were collected in BioEdit (Hall, 1999) and aligned using MAFFT version 7.271 (Katoh et al., 2002; Katoh and Toh, 2008). We employed the Maximum Likelihood (ML) algorithm to construct the phylogenetic tree in MEGA version 11 (Tamura et al., 2021). The molecular model of sequence evolution was determined using Prottest 3.0 (Darriba et al., 2011). To obtain support values at each node, we used 100 bootstrap replicates. Trees were visualized and edited with MEGA version 11.

## Data Availability

The original contributions presented in the study are publicly available. This data can be found here: https://www.ncbi.nlm.nih.gov/geo/query/acc.cgi?acc=GSE334447.

## Supplementary material

SUPPLEMENTARY FIGURE S1 General morphology of *T*. *scripta* embryos at stage 19 (st.19), st.21 and st.23. St.19 embryo in lateral view. St.21 and st.23 embryos in dorsal view.

SUPPLEMENTARY FIGURE S2 (A) Scree plot and **(B)** PCA plot for RNA-seq on peripheral nerve tissues collected from embryos at developmental st.19 (N = 1) and st.21(N=1).

SUPPLEMENTARY FIGURE S3 Gene ontology analysis of **(A)** up-regulated and **(B)** down-regulated genes for RNA-seq on peripheral nerve tissues collected from *T. scripta* embryos at st.19 (N = 1) and st.21 (N = 1).

SUPPLEMENTARY FIGURE S4 Phylogeny of vertebrate *erbB* genes. Unrooted tree of predicted amino acid sequences encoded by vertebrate *erbB* paralogs, including the *erbB-2* gene known to be involved in myelin development, showing that turtles (*Trachemys scripta* and *Chrysemys picta*) possess *bona fide erbB-2* genes. The tree was constructed using the Maximum Likelihood algorithm, and the molecular evolutionary model was JTT + Γ_4_ + I, with 100 bootstrap replicates. See Supplementary Table S4 for species abbreviations.

SUPPLEMENTARY FIGURE S5 Phylogeny of vertebrate *ITGB* genes. Unrooted tree of predicted amino acid sequences encoded by vertebrate *ITGB* paralogs, including the *ITGB4* gene known to be involved in myelin development, showing that turtles (*Trachemys scripta* and *Chrysemys picta*) possess *bona fide ITGB4* genes. The tree was constructed with the Maximum Likelihood algorithm, and the molecular evolutionary model used was JTT + Γ_4_ + I, with 100 bootstrap replicates. See Supplementary Table S4 for species abbreviations.

SUPPLEMENTARY FIGURE S6 Phylogeny of vertebrate *EGR* genes. Unrooted tree of predicted amino acid sequences encoded by vertebrate *EGR* paralogs, including the *EGR2/Krox-20* gene known to be involved in myelin development, showing that turtles (*Trachemys scripta* and *Chrysemys picta*) possess *bona fide EGR2/Krox-20* genes. The tree was constructed with the Maximum Likelihood algorithm, and the molecular evolutionary model used was JTT + Γ_4_ + I, with 100 bootstrap replicates. See Supplementary Table S4 for species abbreviations.

SUPPLEMENTARY FIGURE S7 Phylogeny of vertebrate *Oct* genes. Unrooted tree of predicted amino acid sequences encoded by vertebrate *Oct* paralogs, including the *Oct-6/SCIP* gene known to be involved in myelin development, showing that turtles (*Trachemys scripta* and *Chrysemys picta*) possess *bona fide Oct-6/SCIP* genes. The tree was constructed with the Maximum Likelihood algorithm, and the molecular evolutionary model used was JTT + Γ_4_ + I, with 100 bootstrap replicates. See Supplementary Table S4 for species abbreviations.

SUPPLEMENTARY FIGURE S8 Phylogeny of vertebrate *Siglec* genes. Unrooted tree of predicted amino acid sequences encoded by a subset of vertebrate *Siglec* paralogs, namely, *Siglec-1* (sialoadhesin), *Siglec-2* (CD22), *Siglec-4* (myelin-associated glycoprotein, MAG), and *Siglec-15*, which are known to form a single group inside the *Siglec* gene family (Bornhöfft et al., 2018). Given that *Siglec* members encode a highly variable number of Ig-like domains (ranging from two to sixteen, Bornhöfft et al., 2018), we used the first (V-set Ig-like domain, present in all *Siglecs*) and the second (C2-set, which varies from one in *Siglec-15* to fifteen in *Siglec-1*) Ig-like domains to construct a phylogenetic tree. *Siglec-4*/*MAG* is a gene known to be involved in myelin development, and turtles (*Trachemys scripta* and *Chrysemys picta*) possess *bona fide Siglec-4/MAG* genes. The tree was constructed with the Maximum Likelihood algorithm, and the molecular evolutionary model used was JTT + Γ_4_ + I, with 100 bootstrap replicates. See Supplementary Table S4 for species abbreviations.

SUPPLEMENTARY FIGURE S9 Phylogeny of vertebrate *MBP* genes. Unrooted tree of predicted amino acid sequences encoded by vertebrate *MBP* orthologs, showing that turtles (*Trachemys scripta* and *Chrysemys picta*) possess *bona fide MBP* genes. The tree was constructed with the Maximum Likelihood algorithm, and the molecular evolutionary model used was JTT + Γ_4_, with 100 bootstrap replicates. See Supplementary Table S4 for species abbreviations.

SUPPLEMENTARY FIGURE S10 Phylogeny of vertebrate *MPZL* genes. Unrooted tree of predicted amino acid sequences encoded by vertebrate *MPZL* paralogs, including the *MPZ* gene known to be involved in myelin development, showing that turtles (*Trachemys scripta* and *Chrysemys picta*) possess *bona fide MPZ* genes. The tree was constructed with the Maximum Likelihood algorithm and the molecular evolutionary model used was JTT + Γ_4_, with 100 bootstrap replicates. See Supplementary Table S4 for species abbreviations.

SUPPLEMENTARY FIGURE S11 Phylogeny of vertebrate *EMP* genes. Unrooted tree of predicted amino acid sequences encoded by vertebrate *EMP* paralogs, including the *PMP22* gene known to be involved in myelin development, showing that turtles (*Trachemys scripta* and *Chrysemys picta*) possess *bona fide PMP22* genes. The tree was constructed with the Maximum Likelihood algorithm and the molecular evolutionary model used was JTT + Γ_4_ + I, with 100 bootstrap replicates. See Supplementary Table S4 for species abbreviations.

SUPPLEMENTARY FIGURE S12 Immunostaining of peripheral nerves in *T. scripta* embryos and hatchlings. Immunofluorescence in sciatic nerves using antibodies against different glial and neuronal markers, namely, acetylated tubulin, S100, neurofilament and GFAP, as indicated. Developmental stages are shown in each panel. Nuclei in all samples were counterstained with DAPI, seen in blue or white. Abbreviations: AceTub (acetylated tubulin); GFAP (glial fibrillary acidic protein); NF (neurofilament). The arrows in I and J indicate Schwann cell nuclei. Scale bars are shown in each panel.

SUPPLEMENTARY FIGURE S13 Myelin specializations in adult *T*. *scripta* peripheral nerves. Immunofluorescence of different *T*. *scripta* peripheral nerves with antibodies against MPZ, DRP2, and Kv1.1. **(A,D,G,J,M)** Peripheral nerves marked with an antibody against human MPZ. All nerves show myelin internodes marked with MPZ and observed in a smooth pattern, except the thoracic nerve, which shows some circular unstained spaces. Nodes of Ranvier are observed as unstained spaces between two myelin segments (asterisks). **(B,E,H,K,N)** Peripheral nerves were marked with an antibody against human DRP2. All nerves show patches of expression reminiscent of myelin appositions (arrowheads). Appositions vary in shape, spacing, and distribution among nerves, with thoracic nerves presenting the sparsest pattern. **(C,F,I,L,O)** Peripheral nerves marked with an antibody against Kv1.1. Transverse staining in myelin segments may correspond to Schmidt-Lanterman incisures. Staining of nuclei in **(O)** is unspecific, as it is also observed in negative controls without the primary antibody.

SUPPLEMENTARY FIGURE S14 Epitope conservation for antibodies against MPZ, DRP2, and Kv1.1 proteins in *T. scripta.* Each panel shows a schematic of protein domains at the top as a reference for the position of each epitope and an alignment of proteins from representative sarcopterygian species at the bottom for **(A)** MPZ, **(B)** DRP2, and **(C)** Kv1.1. Alignments include five turtles (highlighted in gray) to show epitope conservation in Testudines. Protein domain models were based on information obtained using the SMART server (Letunic and Bork, 2026; Schultz et al., 1998). Letters on red background indicate amino acids with 100% similarity, red letters on white background indicate regions with 70% or greater similarity, and areas within blue boxes represent important regions that do not tend to vary much. The numbering shown at the beginning and end (red dots) is according to the position of the epitope in *H. sapiens.* Alignments were performed using MAFFT software version 7.271 (Katoh et al., 2002; Katoh and Toh, 2008) and visualized with ESPript 3 (Gouet et al., 1999). Protein domain abbreviations: Broad-Complex, Tramtrack and Bric-à-Brac (BTB); Immunoglobulin V-Type (IGv); Spectrin repeats (SPEC); Signal Peptide (SP); Transmembrane region (TM); Domain with 2 conserved Trp residues (WW); Zinc-binding domain (ZnF). Species abbreviations: Ac, *Anolis carolinensis*; Am, *Ambystoma mexicanum*; Cm, *Chelonia mydas*; Cpb, *Chrysemys picta bellii*; Hs, *Homo sapiens*; Lc, *Latimeria chalumnae*; Mm, *Mus musculus*; Pa, *Protopterus annectens*; Ps, *Pelodiscus sinensis*; Pt, *Pseudonaja textilis*; Tg, *Taeniopygia guttata*; Tmt, *Terrapene mexicana triunguis*; Ts, *Trachemys scripta*.
