# Supplementary figures and images for: Myelin development in the peripheral nervous system of Trachemys scripta
Source: Front Cell Dev Biol. 2026 Jun 18;14:1810247. doi: 10.3389/fcell.2026.1810247 (PMC13324653; doi:10.3389/fcell.2026.1810247)

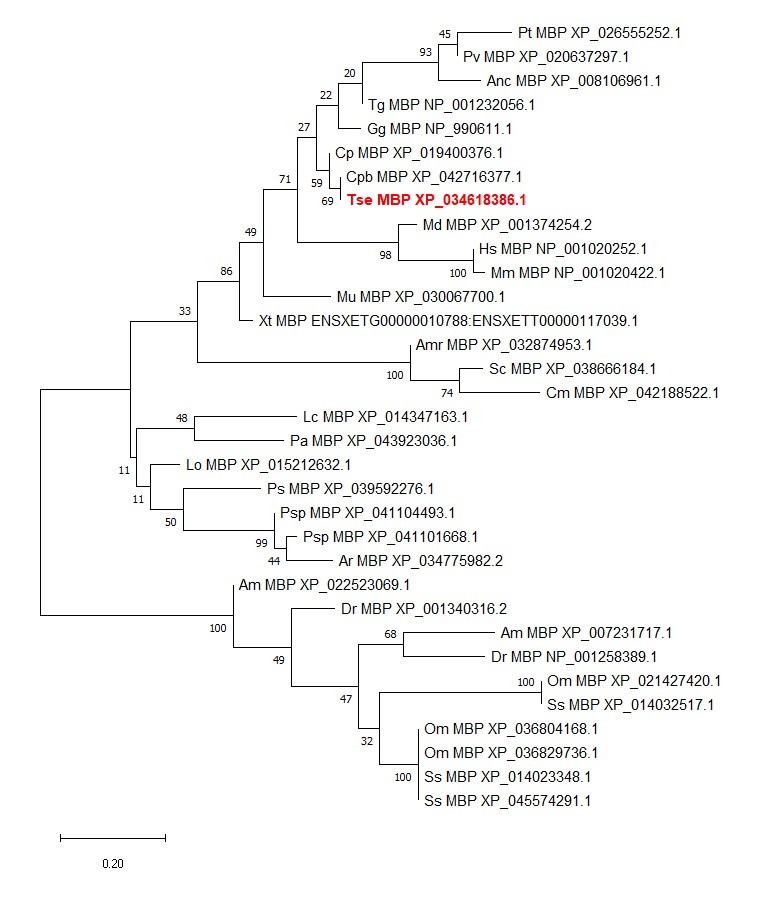

Supplement: Supplementary file 1 [file Image9.tiff]

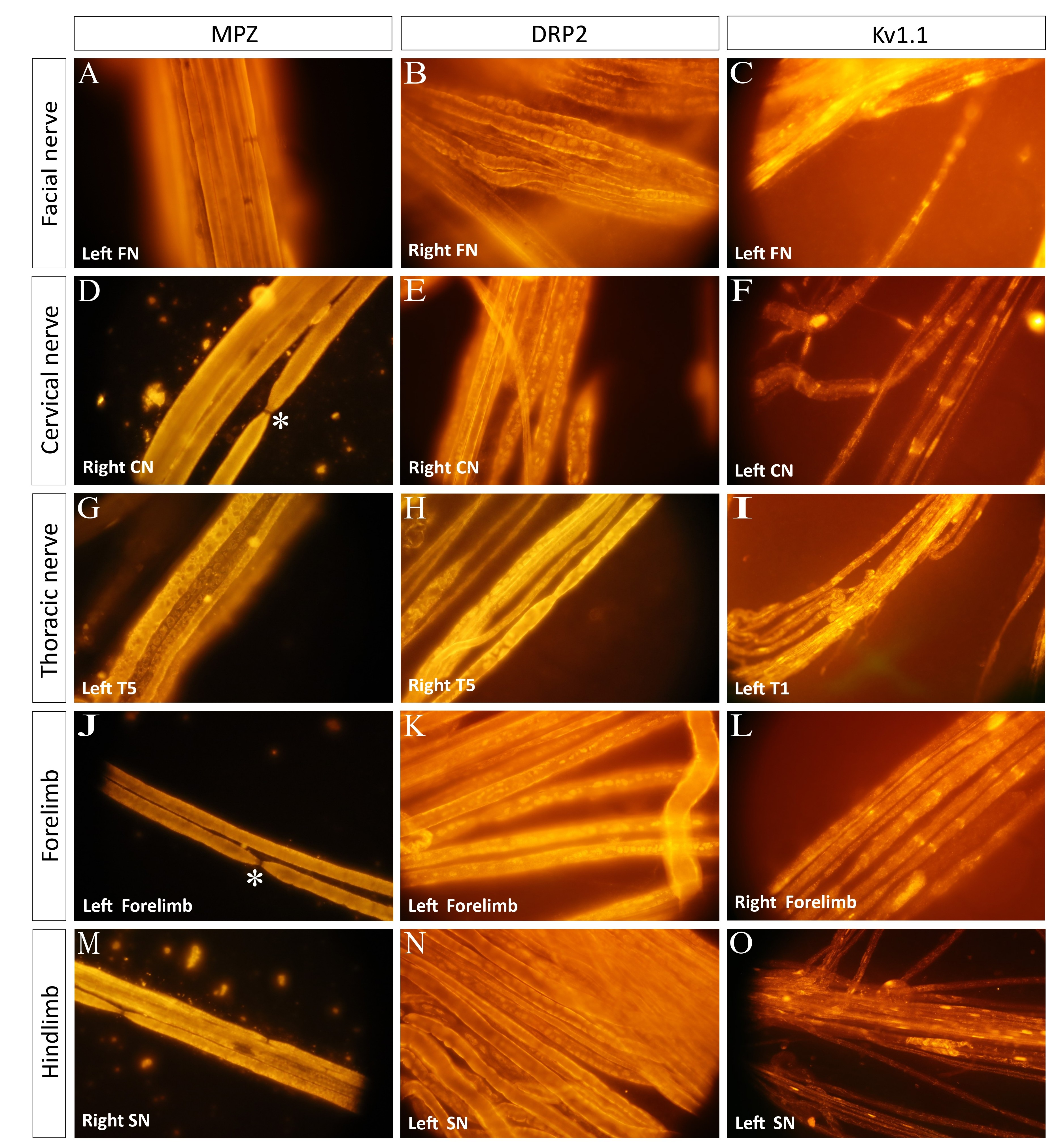

Supplement: Supplementary file 3 [file Image13.tiff]

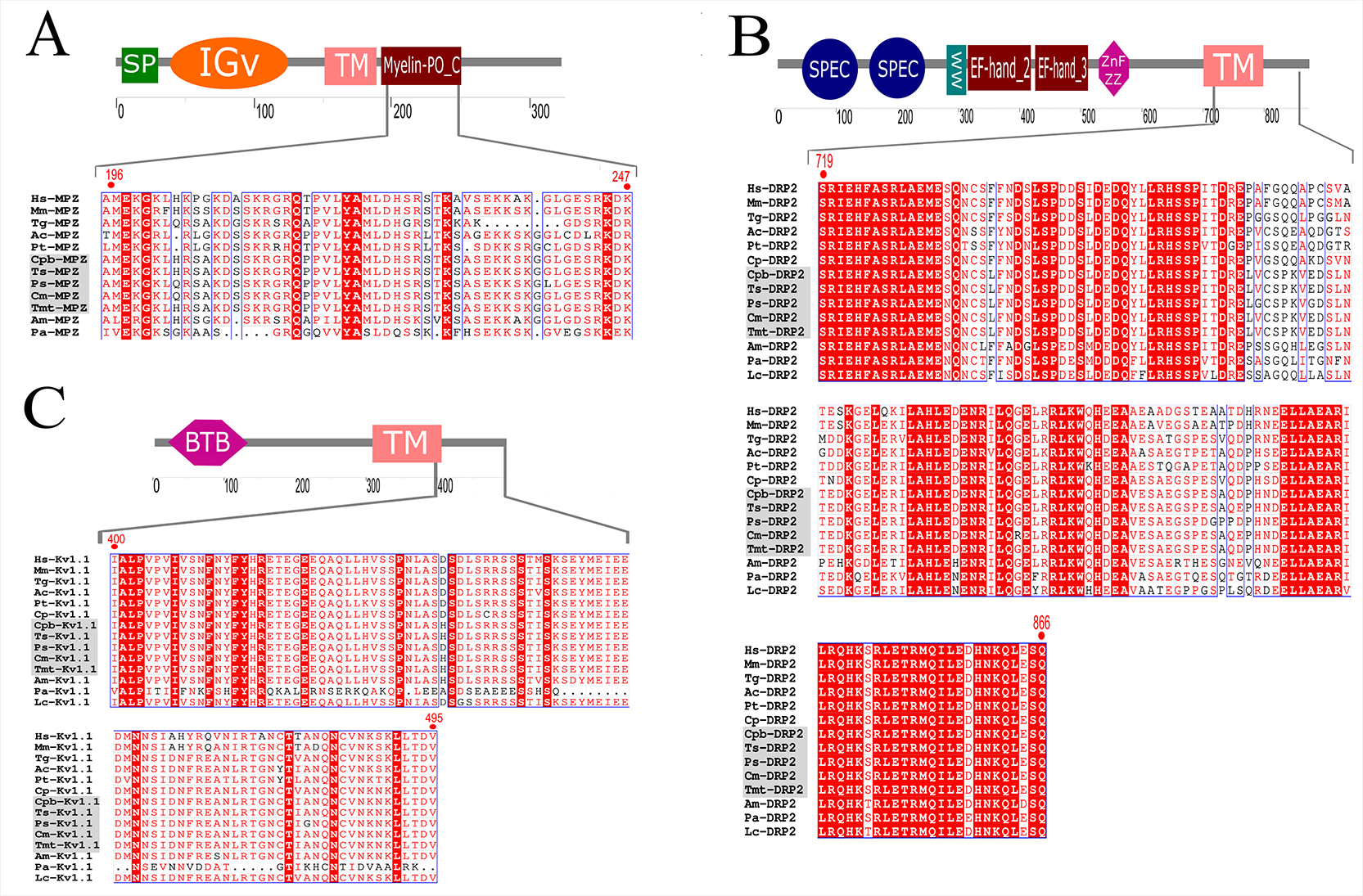

Supplement: Supplementary file 4 [file Image14.tif]

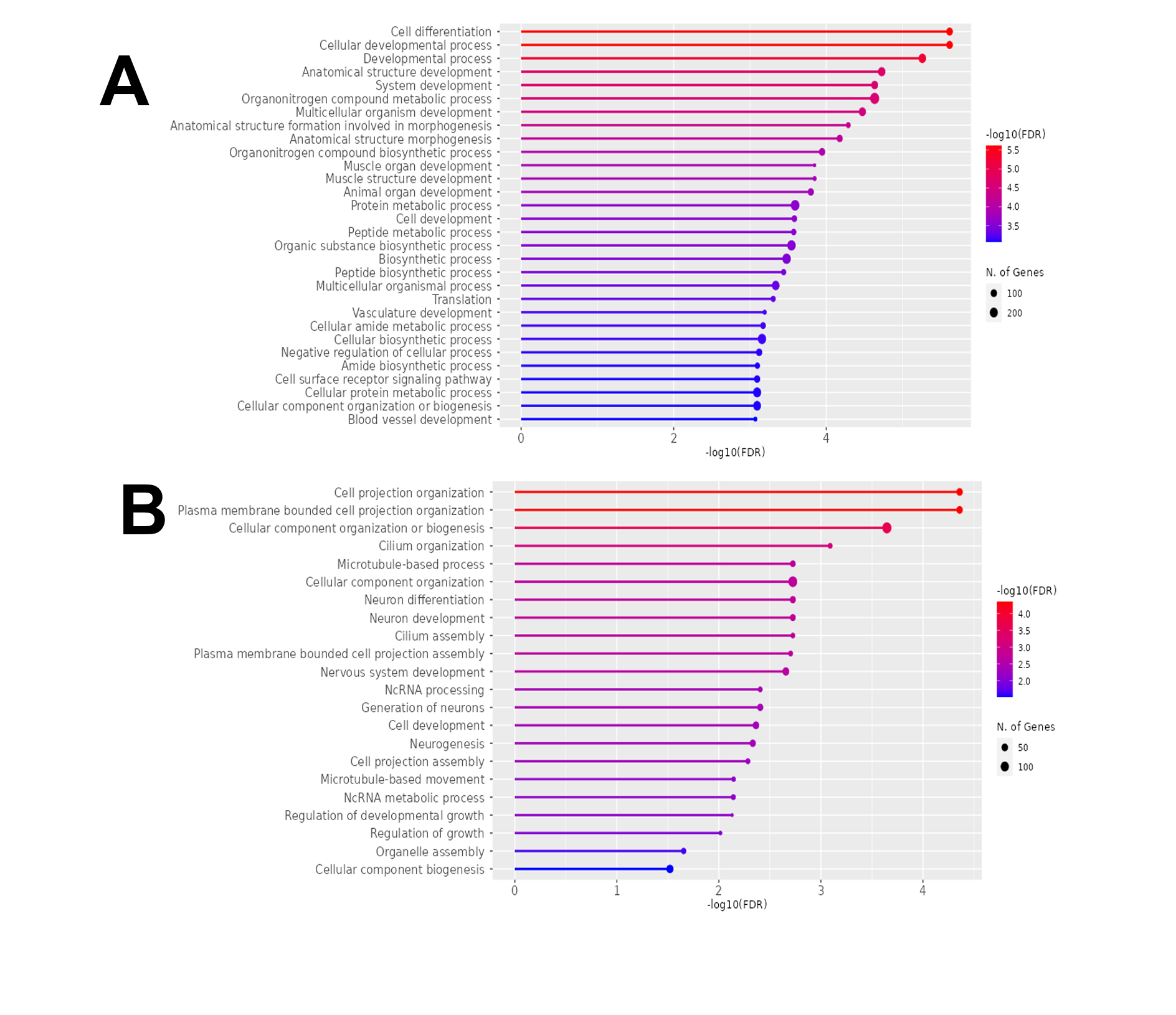

Supplement: Supplementary file 5 [file Image3.tif]

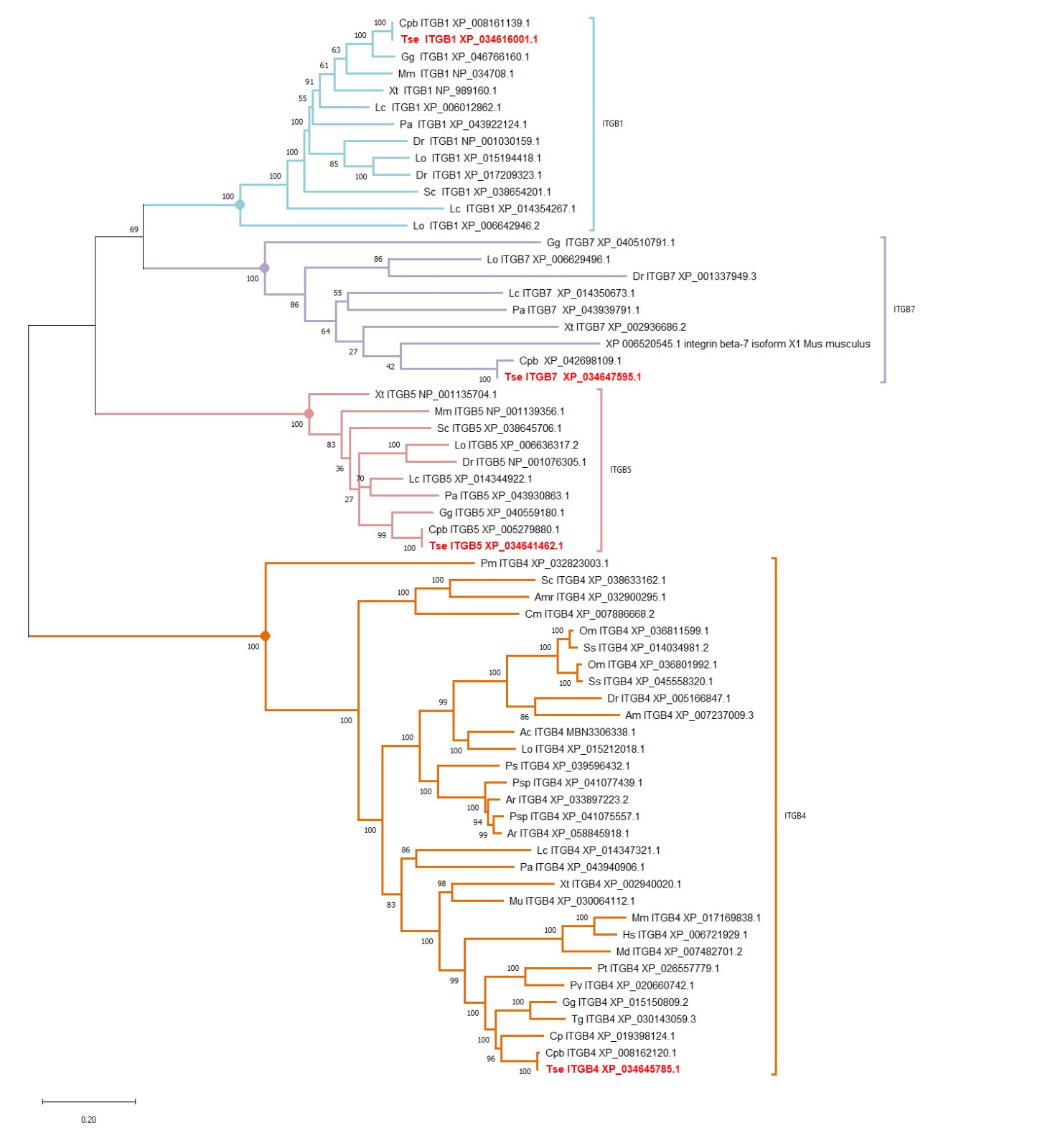

Supplement: Supplementary file 6 [file Image5.tiff]

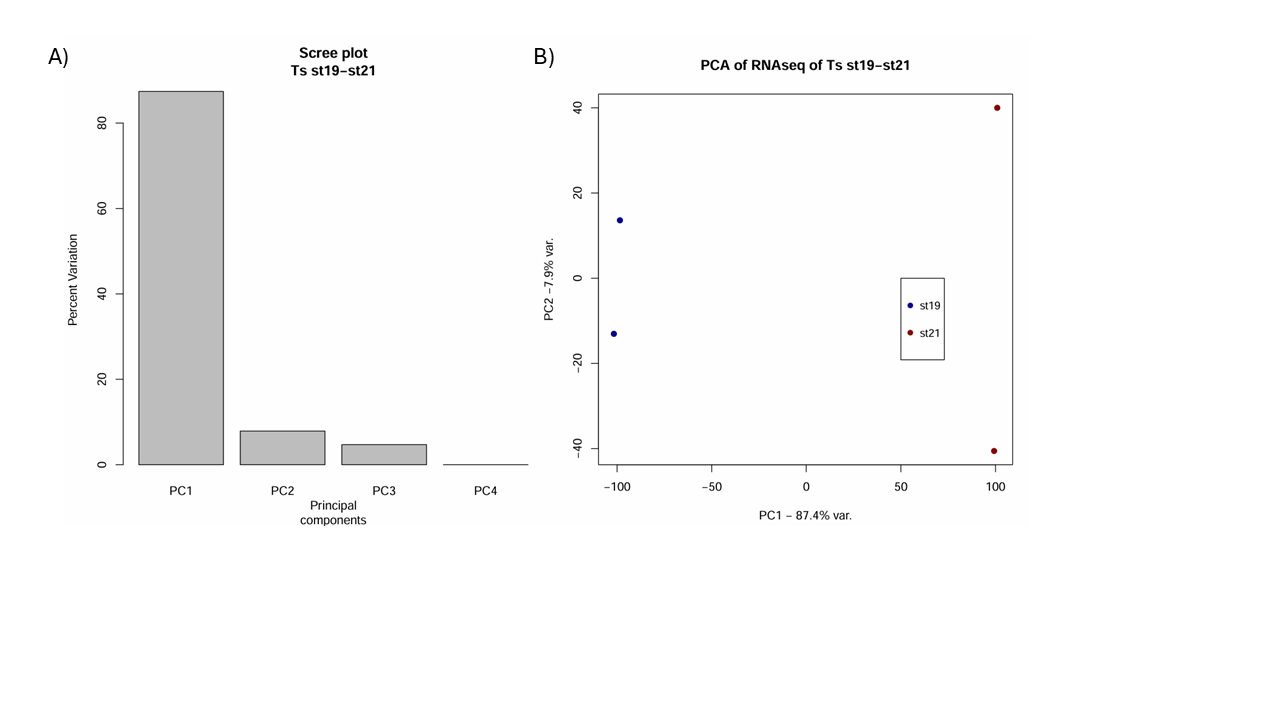

Supplement: Supplementary file 7 [file Image2.tif]

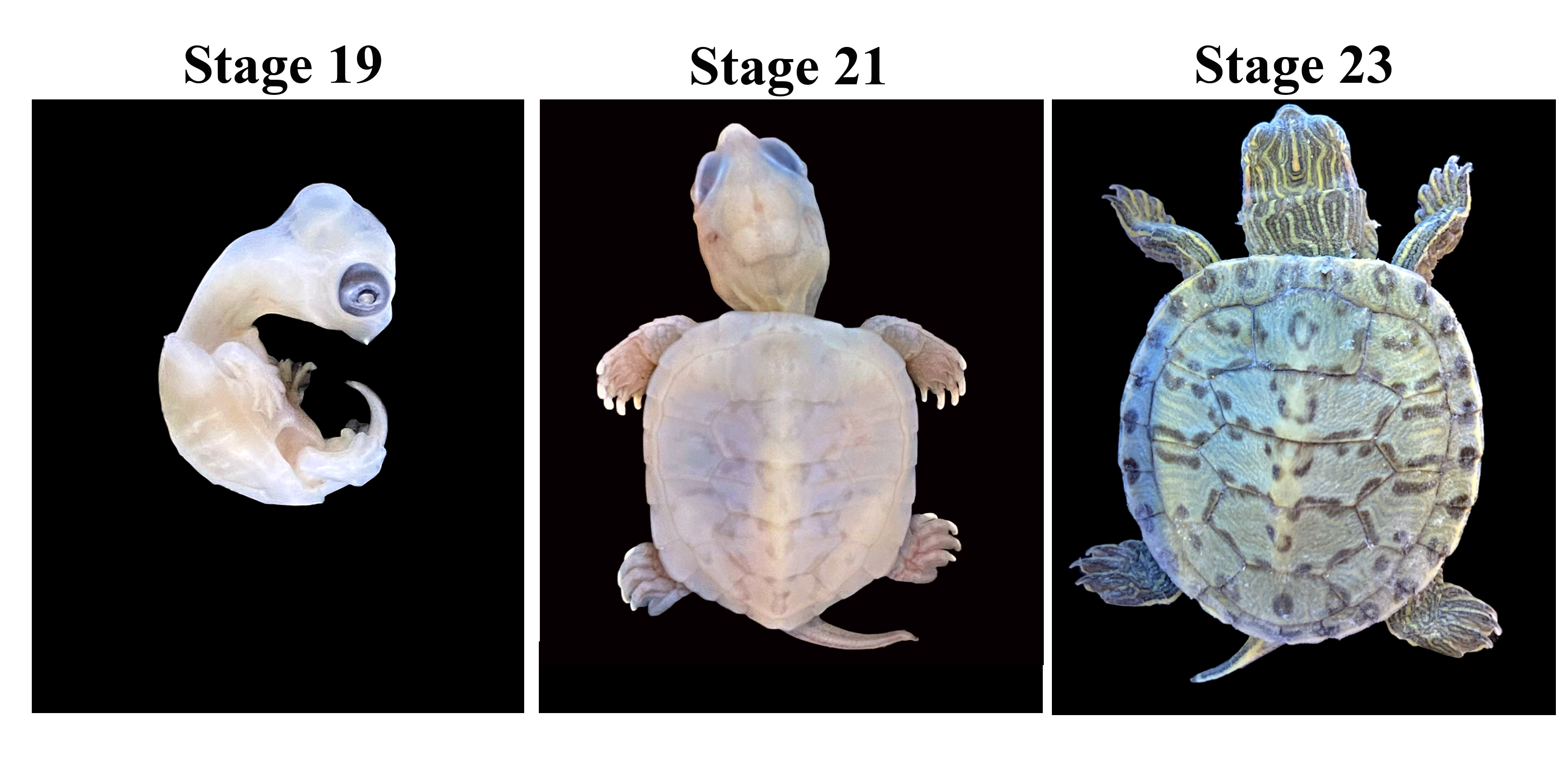

Supplement: Supplementary file 8 [file Image1.tif]

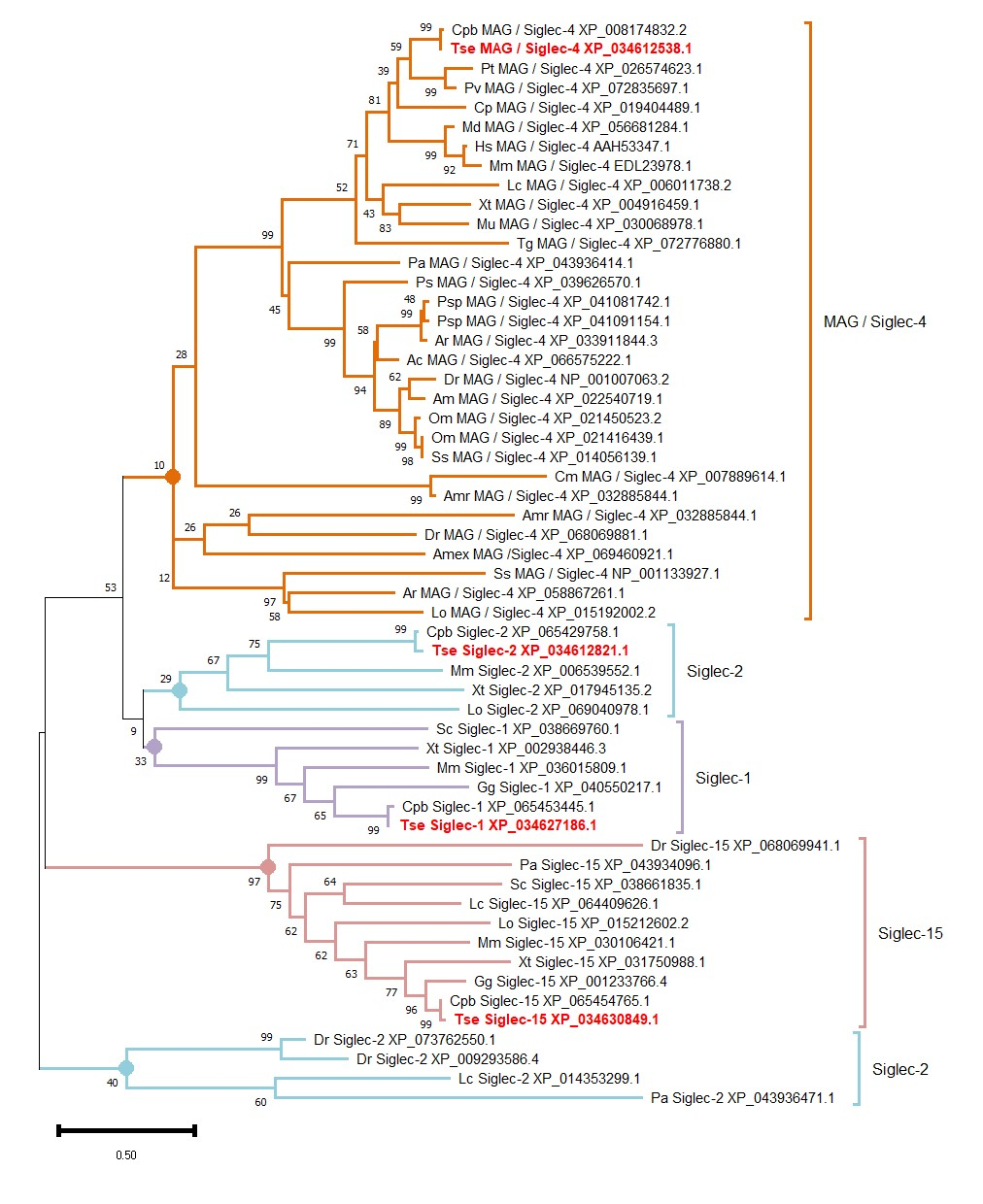

Supplement: Supplementary file 9 [file Image8.tiff]

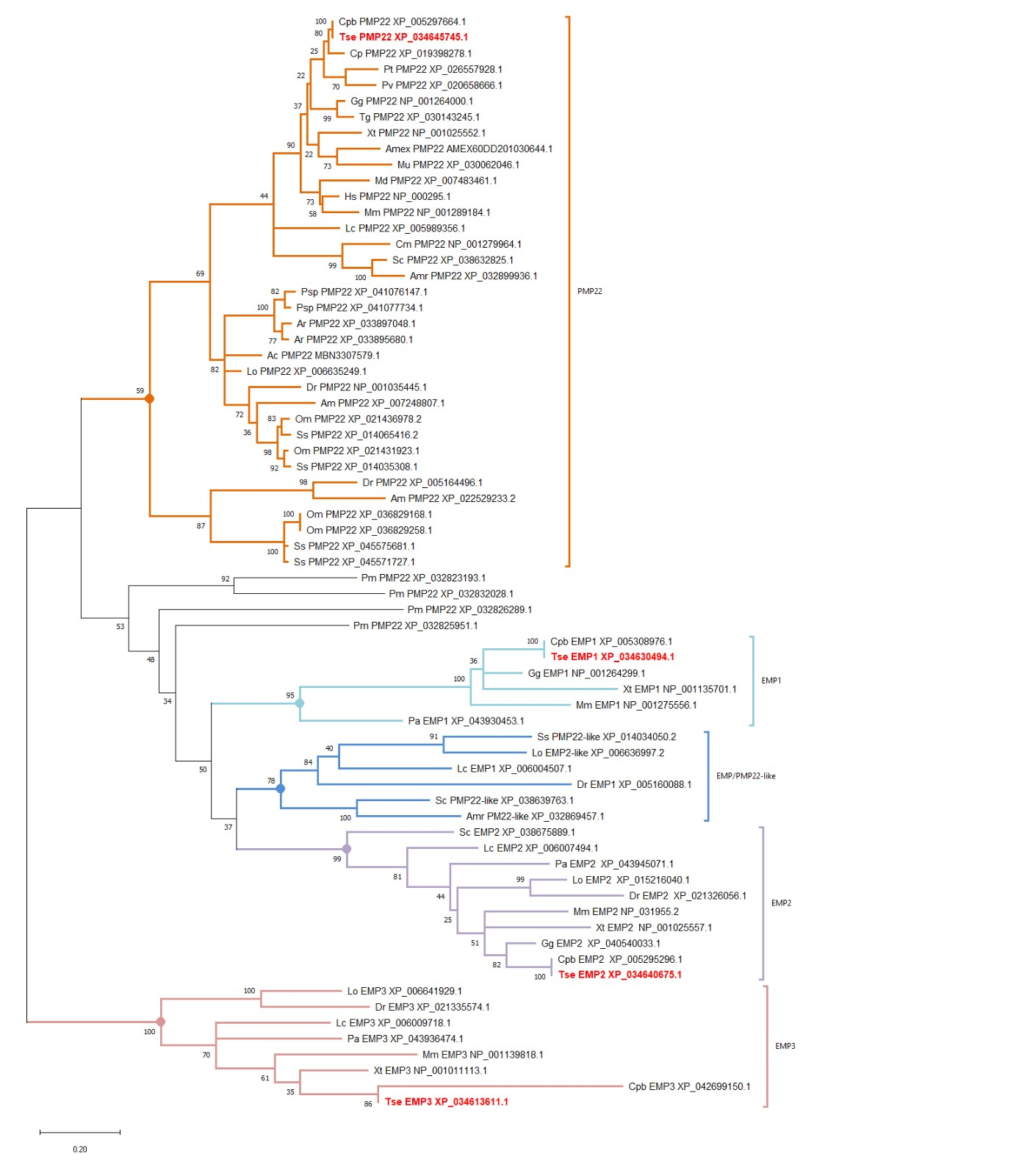

Supplement: Supplementary file 13 [file Image11.tiff]

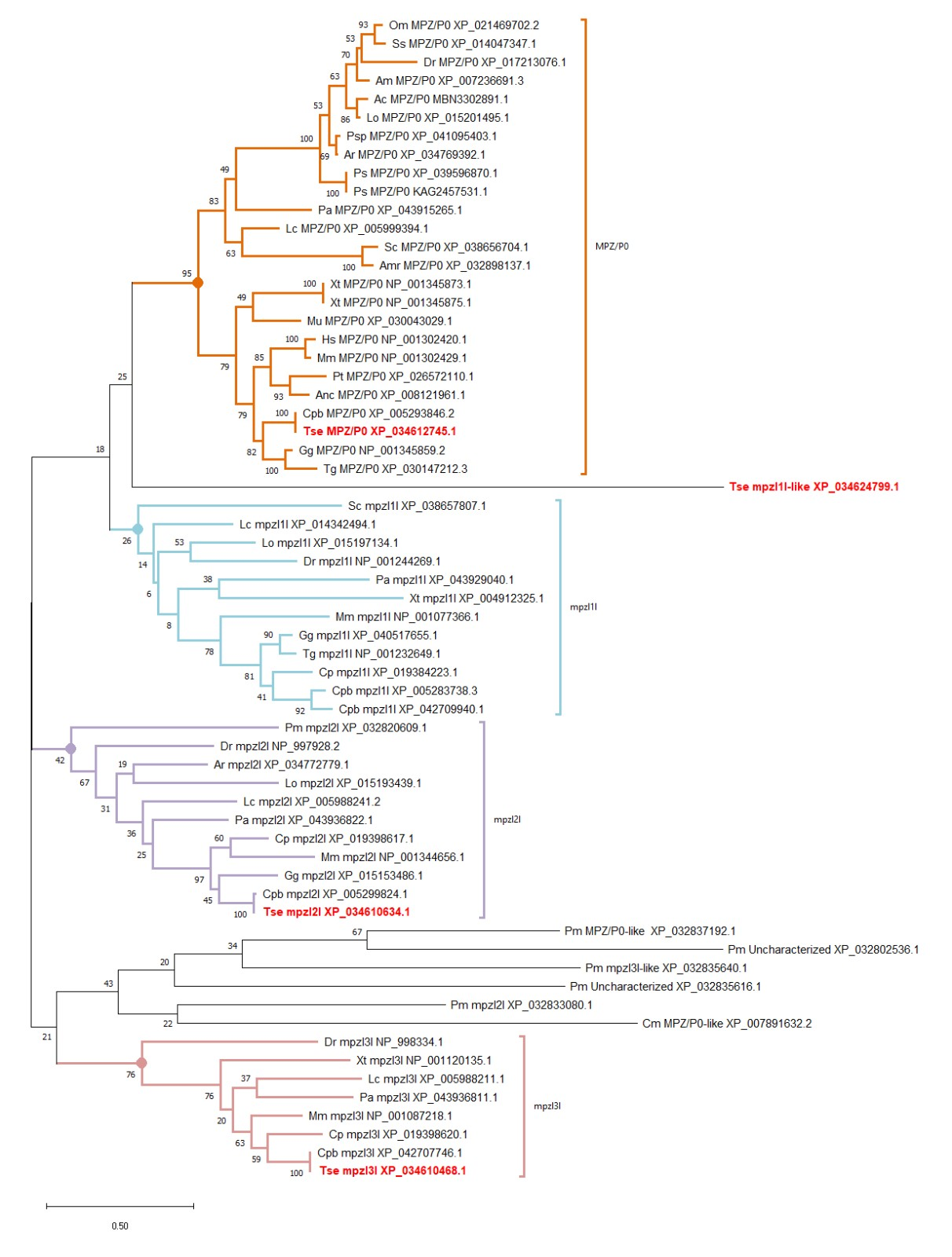

Supplement: Supplementary file 14 [file Image10.tiff]

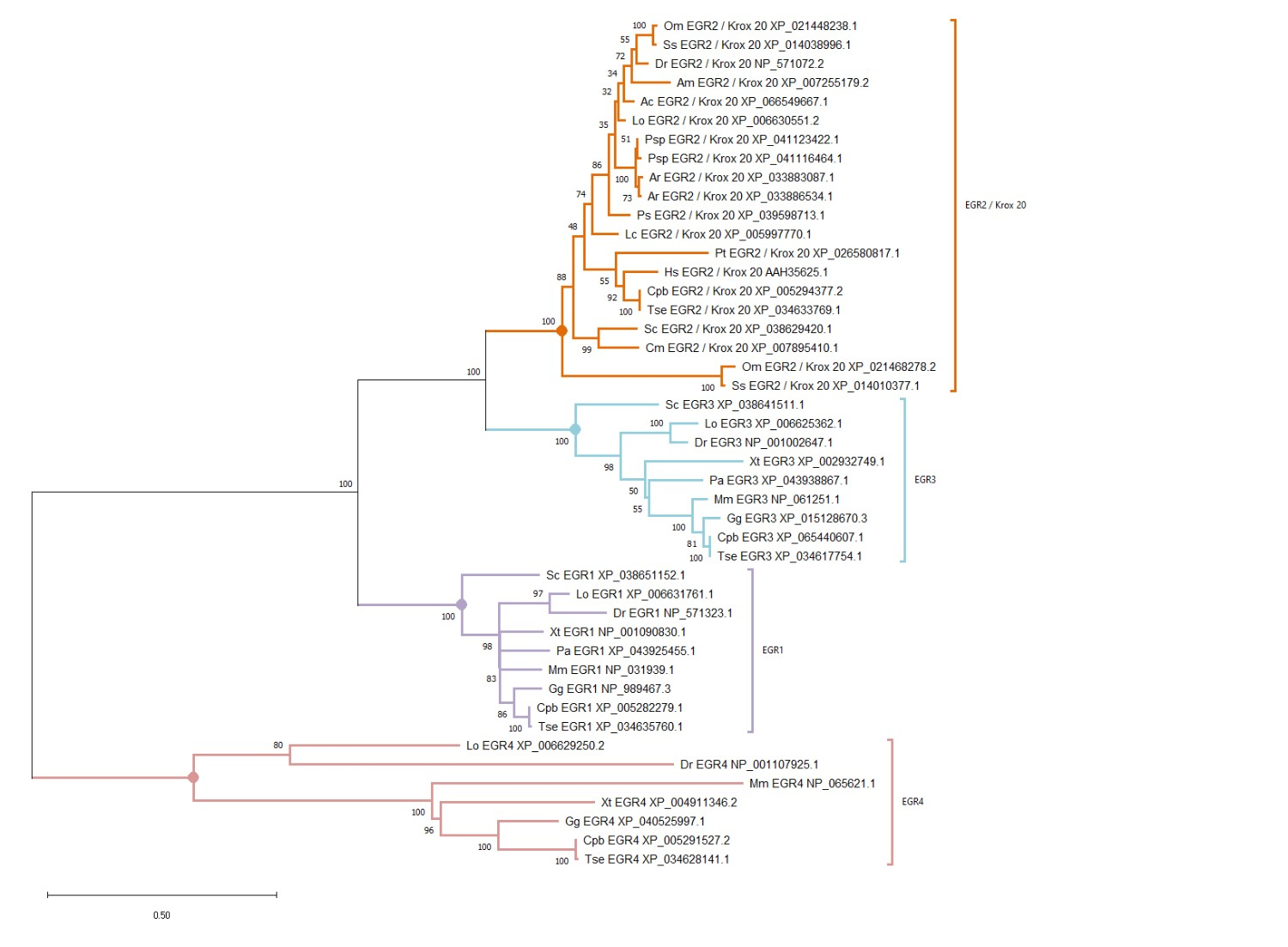

Supplement: Supplementary file 15 [file Image6.tiff]

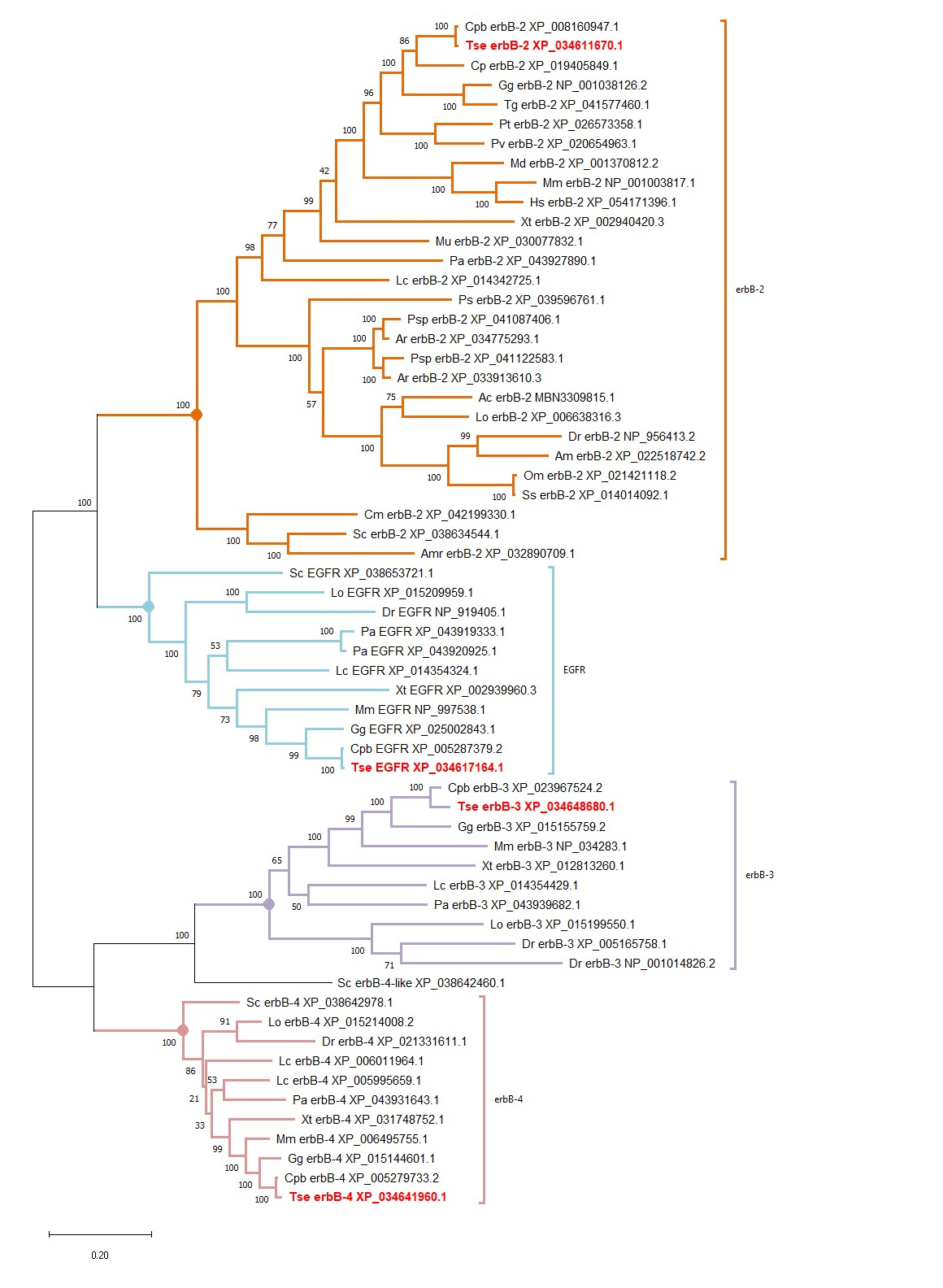

Supplement: Supplementary file 17 [file Image4.tiff]

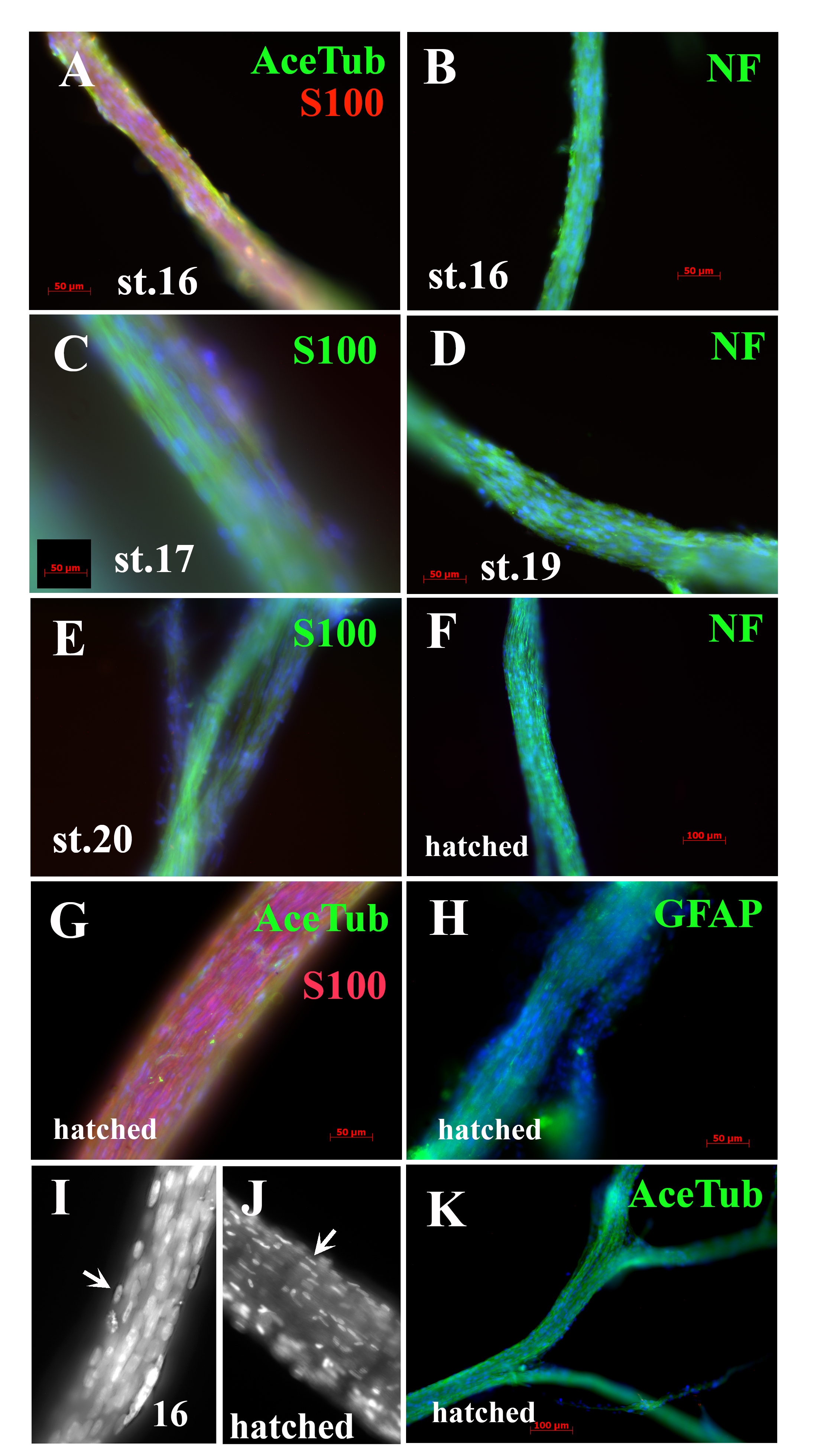

Supplement: Supplementary file 18 [file Image12.tif]

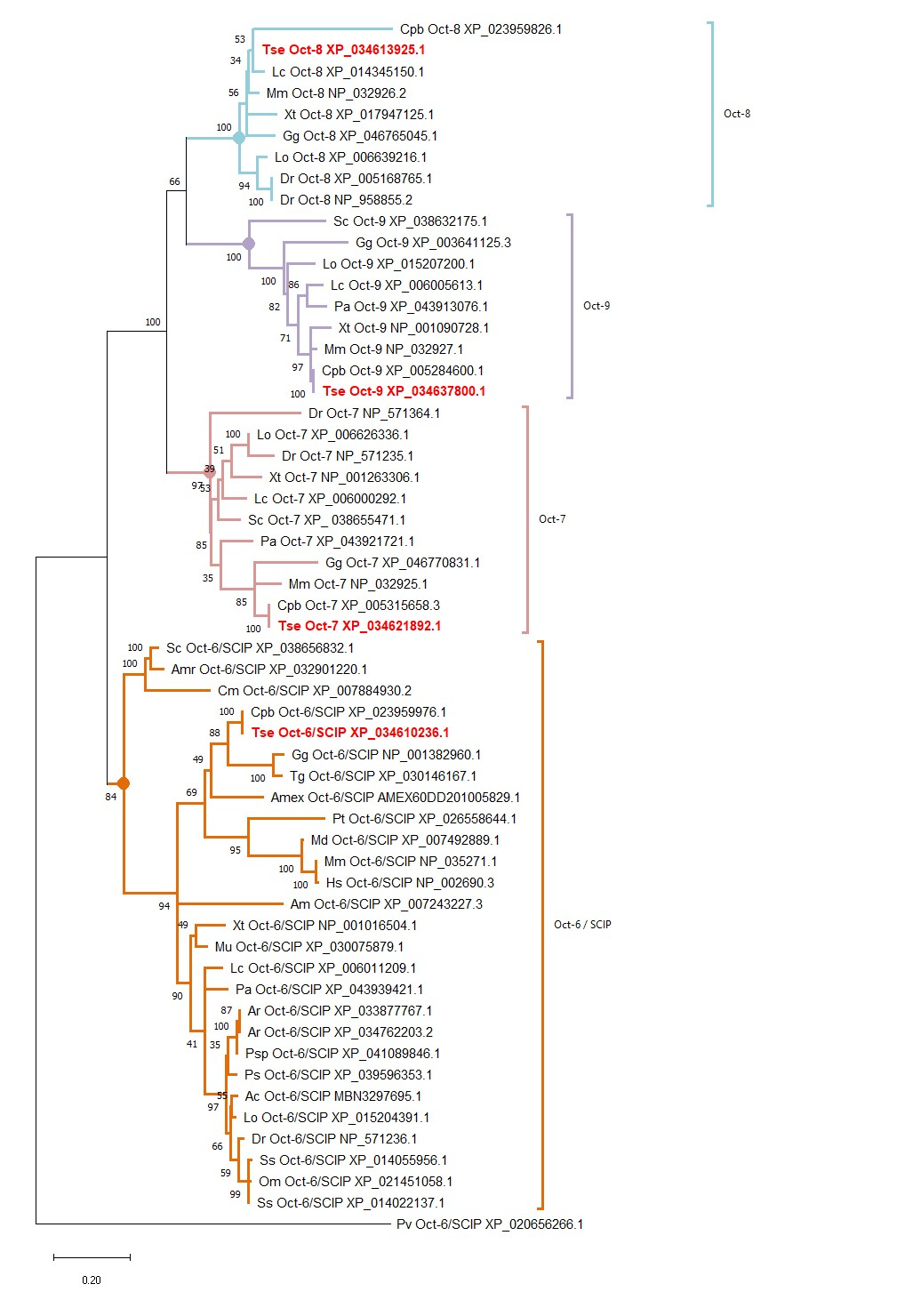

Supplement: Supplementary file 19 [file Image7.tiff]
