# Supplementary material for: Myelin development in the peripheral nervous system of Trachemys scripta
Source: Front Cell Dev Biol. 2026 Jun 18;14:1810247. doi: 10.3389/fcell.2026.1810247 (PMC13324653; doi:10.3389/fcell.2026.1810247)
Supplement: Supplementary file 2 [file Table1.docx]

| ***Table 1: Twenty-five most abundant genes per sample in T. scripta elegans, based on KEGG database.*** *This table shows the twenty-five most abundant genes in the samples that were sequenced by Arraystar, based on a BLAST against the KEGG database.* |
| --- |

| **Stage 19** | | **Stage 21** | |
| --- | --- | --- | --- |
| **Gene** | **Counts** | **Gene** | **Counts** |
| COX1 | 24915 | COL1A | 30953 |
| COL1A | 22164 | COX1 | 20847 |
| TubB | 13988 | Actb_g1 | 6916 |
| Actb_g1 | 12526 | TubB | 6693 |
| TubA | 10857 | TubA | 6144 |
| MAP1 | 8695 | COX2 | 5319 |
| DYNC1H | 8144 | EEF1A | 5197 |
| EEF2 | 6773 | ND1 | 5082 |
| SPTB | 6548 | EEF2 | 4460 |
| EEF1A | 6515 | FLNA | 4004 |
| COX2 | 5795 | HSPG2 | 3957 |
| YWHAE | 5751 | ND5 | 3483 |
| FLNA | 5454 | H2A | 3336 |
| ND1 | 5357 | H3 | 3019 |
| HSP90A | 4782 | SPTB | 2858 |
| ND5 | 4710 | MAP1 | 2849 |
| KIF1 | 4380 | DYNC1H | 2828 |
| ANK | 4342 | YWHAE | 2749 |
| HSPG2 | 3851 | COX3 | 2731 |
| KRAB | 3807 | CYTB | 2369 |
| FASN | 3612 | HSP90A | 2357 |
| COL18A | 3459 | COL6A | 2347 |
| SPTA | 3201 | COL18A | 2160 |
| NOTCH1 | 3191 | NOTCH1 | 2141 |
| CYTB | 3184 | NID | 2123 |
