# Supplementary material for: Myelin development in the peripheral nervous system of Trachemys scripta
Source: Front Cell Dev Biol. 2026 Jun 18;14:1810247. doi: 10.3389/fcell.2026.1810247 (PMC13324653; doi:10.3389/fcell.2026.1810247)
Supplement: Supplementary file 10 [file Table2.docx]

***Table 2: Overview of the abundance of a selection of markers per sequenced RNA sample.*** *This table shows the RNA sequencing results for a selection of genes related to myelination, including the position in the gene list (in which place the gene ended) and the FPKM. These results are based on the BLAST against the corresponding genomes downloaded from Genbank.*

|  | **Stage 19** | | **Stage 21** | | **Stage 21** | |
| --- | --- | --- | --- | --- | --- | --- |
| **Gene** | **Position in gene list** | **FPKM** | **Position in gene list** | **FPKM** | **Position in gene list** | **FPKM** |
| AP2A1 | 2560 | 6 | 2351 | 4 | 939 | 9 |
| CDH19 | 1386 | 10 | 919 | 8 | 2403 | 4 |
| CNP | 5778 | 3 | 4659 | 2 | 4831 | 2 |
| FOXD3 | 507 | 23 | 372 | 18 | 322 | 24 |
| GAPDH | 106 | 92 | 110 | 65 | 100 | 83 |
| GFAP | 7262 | 2 | 3281 | 3 | 2534 | 3 |
| ITGB4 | 12342 | < 1 | 10906 | < 1 | 9867 | < 1 |
| KROX20 | 10148 | 1 | 7463 | 1 | 7680 | < 1 |
| MAG | 14293 | < 1 | 9139 | < 1 | 6737 | 1 |
| MBP | 556 | 21 | 202 | 37 | 404 | 19 |
| MPZ | 151 | 72 | 81 | 89 | 66 | 113 |
| PLP1 | 258 | 26 | 260 | 26 | 262 | 30 |
| PMP22 | 376 | 31 | 219 | 33 | 320 | 24 |
| SCIP | 15657 | < 1 | 15887 | < 1 | 14416 | < 1 |
| SOX10 | 587 | 20 | 482 | 15 | 423 | 19 |
