# Supplementary material for: Myelin development in the peripheral nervous system of Trachemys scripta
Source: Front Cell Dev Biol. 2026 Jun 18;14:1810247. doi: 10.3389/fcell.2026.1810247 (PMC13324653; doi:10.3389/fcell.2026.1810247)
Supplement: Supplementary file 12 [file Table3.docx]

| ***Supplementary Table 1: Overview of the of primers for T. scripta elegans genes and the expected length of the corresponding product.*** *The primers were based on the sequencing results of the products amplified with the primers from batch one and were designed using Geneious v11.4.1.* | | | | |
| --- | --- | --- | --- | --- |
| **Name primer F** | **Sequence primer F** | **Name primer R** | **Sequence primer R** | **Target length** |
| qMPZ F | GTACGCCATGCTGGACCATA | qMPZ R | TTCTTGTCCTTGCGGGACTC | 90 |
| qMBP F | CAGTCCATTGCTGGGACCTT | qMBP R | TATGACTGGGACAGCAGGGA | 99 |
| qMAG F | ACAGCAACCTGGAGATCATCG | qMAG R | ATGGTGGGGCAGTTATCCG | 109 |
| qerbB2 F | GCCAAGGCAGCTTGGATATG | qerbB2 R | GTTACGGGCCTTGGTGCTAT | 100 |
| qKrox20 F | TCAGGGCCTCCCCATAAATAC | qKrox20 R | AGCCCAGTAGTTGCTGTAGT | 118 |
| qPMP22 F | CAGTGTAACMAGAGCGCAGT | qPMP22 R | GGGTTTCTTTGGTGGGTTGC | 109 |
| qITGB4 F | AAGGCTGAGGGTAGTAGGGG | qITGB4 R | GCTAGGATGCCTCATCGACC | 106 |
| qSCIP F | TCAAGCCCCTGCTCAACAAA | qSCIP R | TCMATGGAGGTGCGCTTCTT | 115 |
| qGAPDH F | CTGAGGGCAAGGTCATTCCG | qGAPDH R | CGACAAGTCAGGTCCACGAC | 97 |
